# A micron-scale surface topography design reducing cell adhesion to implanted materials

**DOI:** 10.1038/s41598-018-29167-2

**Published:** 2018-07-18

**Authors:** Francesco Robotti, Simone Bottan, Federica Fraschetti, Anna Mallone, Giovanni Pellegrini, Nicole Lindenblatt, Christoph Starck, Volkmar Falk, Dimos Poulikakos, Aldo Ferrari

**Affiliations:** 1 Laboratory of Thermodynamics in Emerging Technologies, Department of Mechanical and Process Engineering, ETH Zurich, Sonneggstrasse 3, CH-8092 Zurich, Switzerland; 2 Wyss Zurich, ETH Zurich/University of Zurich, Zurich, Switzerland; 3 Institute for Regenerative Medicine, University of Zurich, Zurich, Switzerland; 4 Laboratory for Animal Model Pathology, Institute of Veterinary Pathology, Vetsuisse Faculty, University of Zurich, Zurich, Switzerland; 5 Department of Plastic and Hand Surgery, University Hospital Zurich, Zurich, Switzerland; 6 Department of Cardiothoracic and Vascular Surgery, German Heart Institute Berlin, Berlin, Germany

## Abstract

The micron-scale surface topography of implanted materials represents a complementary pathway, independent of the material biochemical properties, regulating the process of biological recognition by cells which mediate the inflammatory response to foreign bodies. Here we explore a rational design of surface modifications in micron range to optimize a topography comprised of a symmetrical array of hexagonal pits interfering with focal adhesion establishment and maturation. When implemented on silicones and hydrogels *in vitro*, the anti-adhesive topography significantly reduces the adhesion of macrophages and fibroblasts and their activation toward effectors of fibrosis. In addition, long-term interaction of the cells with anti-adhesive topographies markedly hampers cell proliferation, correlating the physical inhibition of adhesion and complete spreading with the natural progress of the cell cycle. This solution for reduction in cell adhesion can be directly integrated on the outer surface of silicone implants, as well as an additive protective conformal microstructured biocellulose layer for materials that cannot be directly microstructured. Moreover, the original geometry imposed during manufacturing of the microstructured biocellulose membranes are fully retained upon *in vivo* exposure, suggesting a long lasting performance of these topographical features after implantation.

## Introduction

The non-specific adhesion of cells and tissues to synthetic substrates is at the origin of adverse responses to body implants, including those associated with foreign body reaction^1^. In a significant percentage of patients, the progression of these processes is rapid and leads to a premature failure of the implanted device^2^. Yet, even mild adhesion to the surgical pocket represents a compelling obstacle to revision surgeries. Complications upon the programmed exchange of cardiac implantable electronic devices (CIEDs) are such an example. In this case, the level of fibrotic adhesion correlates with an increased risk of secondary infections, lead damage, and bleeding^3^. A strategy to hinder cell and tissue adhesion to artificial materials would therefore provide the significant benefit of avoiding unplanned revisions and facilitating the scheduled exchange of implanted devices. A universal anti-adhesive solution is however still missing and represents an unmet need in current implant technology.

The exposure of implanted biomaterial interfaces to cell adhesion is determined by the physic-chemical properties of the synthetic surface^4,5^. In particular, the hydrophilicity, rigidity, and topography of a planar substrate contribute to define its potential interaction with cells^6^. While the aforementioned surface parameters can be independently tailored by means of chemical functionalization or processing^7^, the overall selection of biomaterials is primarily dictated by the implant architecture and function.

Silicones are commonly used to manufacture aesthetic implants or to isolate conductive elements of CIEDs. The formulation of such elastomers offers high processability and yields substrates featuring a broad range of deformability, resilience, and toughness^8^. Yet, silicon-based interfaces (e.g. drivelines of ventricular assist devices VADs, pacemakers, gastric and deep brain stimulators, breast implants) generate hotspots for the onset of fibrotic responses upon deployment^9^. To mitigate this problem, several surface treatments have been tested, including modification of roughness and chemistry. Modification of artificial substrates with nano-scale topographies has shown the ability to reduce protein absorption^10^. These non-fouling surfaces can demote cell adhesion blocking integrin-mediated surface recognition. However, anti-fouling treatments may have limited durability when exposed to mechanically-challenging environments and biological fluids *in vivo*. To date, none of these solutions have given satisfactory results *in vivo*.

A promising alternative approach is represented by the application of an intervening protective layer between the device and the hosting tissue. In this direction, bio-synthesized cellulose (i.e. biocellulose) is attracting growing interest due to its favorable properties, which include the long-term stability and the low inflammatory response elicited *in vivo*^11–14^. In addition, the bacterial fermentation process generating biocellulose offers an easy access to the modification of several material properties such as density^15^, chemistry^16^, and surface topography^17^.

Micron-scale surface topography is an independent parameter that can be modified without affecting the bulk mechanical or chemical properties of a target substrate. The performance of surface geometries in this length-scale in inducing specific cellular responses is well established^18^. Specifically, a rational design of regular topographic features can reduce the adhesion, spreading and/or the differentiation of several cell types, including fibroblasts, inflammatory precursors, and others. Examples of such geometries include dots, cones, pyramids, pits, funnels, circular elements, and inverted pyramids^19–23^. The biological mechanism responsible for the anti-adhesive properties is based on the direct physical interference of topographical elements with the process of focal adhesion establishment and maturation during cell spreading. Micron-scale isotropic elements arranged in a symmetric and regular pattern can physically block the process of spreading and elongation. Importantly, geometrical elements of this size are still sensed by cells upon coating with extracellular matrix (ECM) proteins^17^. In addition to the performance in inducing the desired biological effect, the rational design of a topography rendering a substrate non-adhesive to cells must consider its scalability to large surfaces and its transferability to the material of choice.

Here, we design and validate an anti-adhesive surface texture topography comprised of regular symmetric arrays of microscale pits in the range between 3 and 20 µm in diameter and center-to-center distances of between 6 and 23 µm. We establish facile and scalable protocols for its high-fidelity transfer on to the surface of silicones or biocellulose substrates. Finally, we demonstrate its ability *in vitro* to demote the adhesion and activation of primary human fibroblasts and macrophages.

## Results

### Generation of anti-adhesive topographies

Topographic features in the submicron and micron range physically interfere with the establishment and maturation of focal adhesions, thus affecting the processes of cell spreading and adhesion to a substrate^24^. In particular, anisotropic geometries (e.g. gratings) promote cell polarization^25^, while isotropic arrays demote the adhesion and differentiation of mammalian cells^19,26^.

To establish a universal anti-adhesive topography, a number of topographic patterns were investigated in the study. The criteria for selection encompassed the biological effect, the ability to transfer the topographical elements on the target materials (i.e. silicones and biocellulose) through established lithography protocols, and the ability to upscale the process to large surfaces. To maximize the resulting biological effect, individual topographic elements were chosen in the micron-scale to interfere with the establishment and maturation of focal adhesions. The surface arrangement of these features was set to be completely symmetrical and isotropic, thus eliminating any preferential direction of focal adhesion establishment^27^. Based on this consideration, the parametric space was adapted to the fabrication limits, altogether setting the separation between topographical elements to 3 μm.

**Figure 1:**
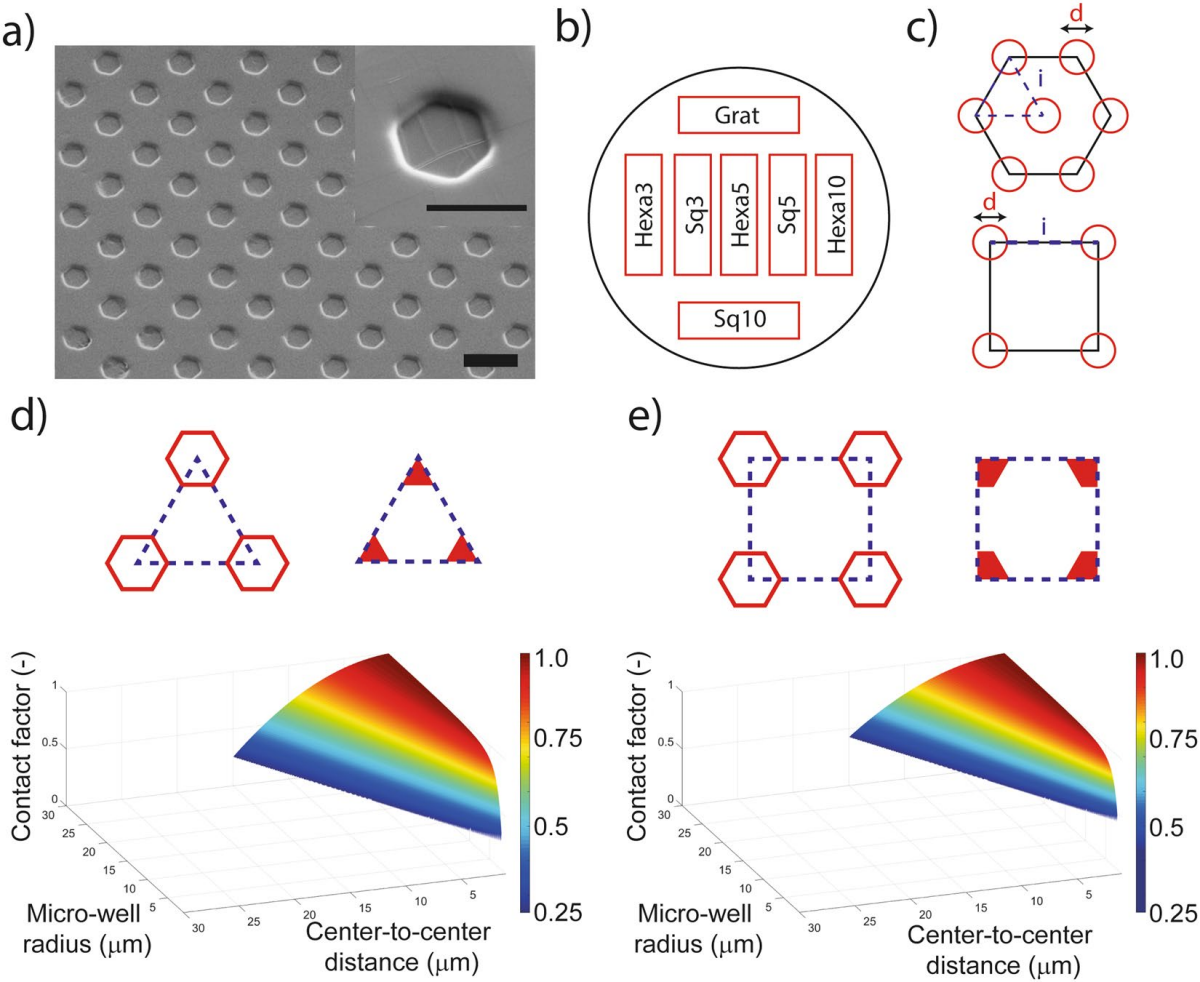
Replica molding and micro-pattern characterization. (**a**) SEM micrograph of elastomeric microstructured substrate manufactured using soft-lithography and coated with fibronectin. Scale bar: 10 µm. Inset, scale bar: 5 µm. (**b**) Layout of the patterns on the elastomeric microstructured substrate for cell adhesion experiments. (**c**) Elemental cells of the investigated patterns for cell adhesion reduction. Parametric design space for elastomeric microstructured substrate in the (**d**) hexagonal and (**e**) squared patterns considering manufacturing constraints and rationale mechanobiological design principles.

The tested geometries featured hexagonal pits and were defined by the three topographic parameters: first, the diameter *d* of the pits, which ranged between 3 and 20 μm (Fig. 1). Second, the inter-element distance *i* which ranged between 6 and 23 μm. Third, the shape of the elemental cell, chosen to be perfectly isotropic (centered-hexagonal, “Hexa”) or quasi-isotropic (squared, “Sq”). In total 12 different patterns were fabricated. The parameters *d* and *i* can be used to define a shape descriptor of the surface called contact factor, defining the amount of flat surface available for cell contact in each configuration. Simple analytical solutions can be found for the contact factor in both configurations:

$$C{F}_{Hexa}=1-\frac{6}{4}\frac{{d}^{2}}{{i}^{2}}\,\cos (60)\,C{F}_{Sq}=1-\frac{4}{3}\frac{{d}^{2}}{{i}^{2}}\,\sin (60)\cos (60)$$

These equations generate two parametric spaces within which the selection is practically limited by geometrical and fabrication constrains (Fig. 1d,e). The effects of prospective anti-adhesive topographies were compared to the ones obtained on identical flat substrates and on gratings with ridge and grove width of 5 μm and grove depth of 1.4 μm.

The surface geometries were initially transferred on silicones (i.e. PDMS) by means of soft lithography. The resulting surface morphology was systematically characterized after fabrication (Supp. Figs 1 and 2) by means of scanning electron microscopy (SEM). Samples were coated with gelatin or fibronectin^28^ for the cell experiments. Importantly, the coating procedures did not alter the geometry of topographic features. In particular, no fibrillar structures were detected on the PDMS substrates (Fig. 1a) and the coating appeared to be conformal and homogeneously distributed on the whole substrate, including the sidewalls and the bottom of the pits (Supp. Video 1).

### Screening of anti-adhesive performance upon interaction with HDFs

**Figure 2:**
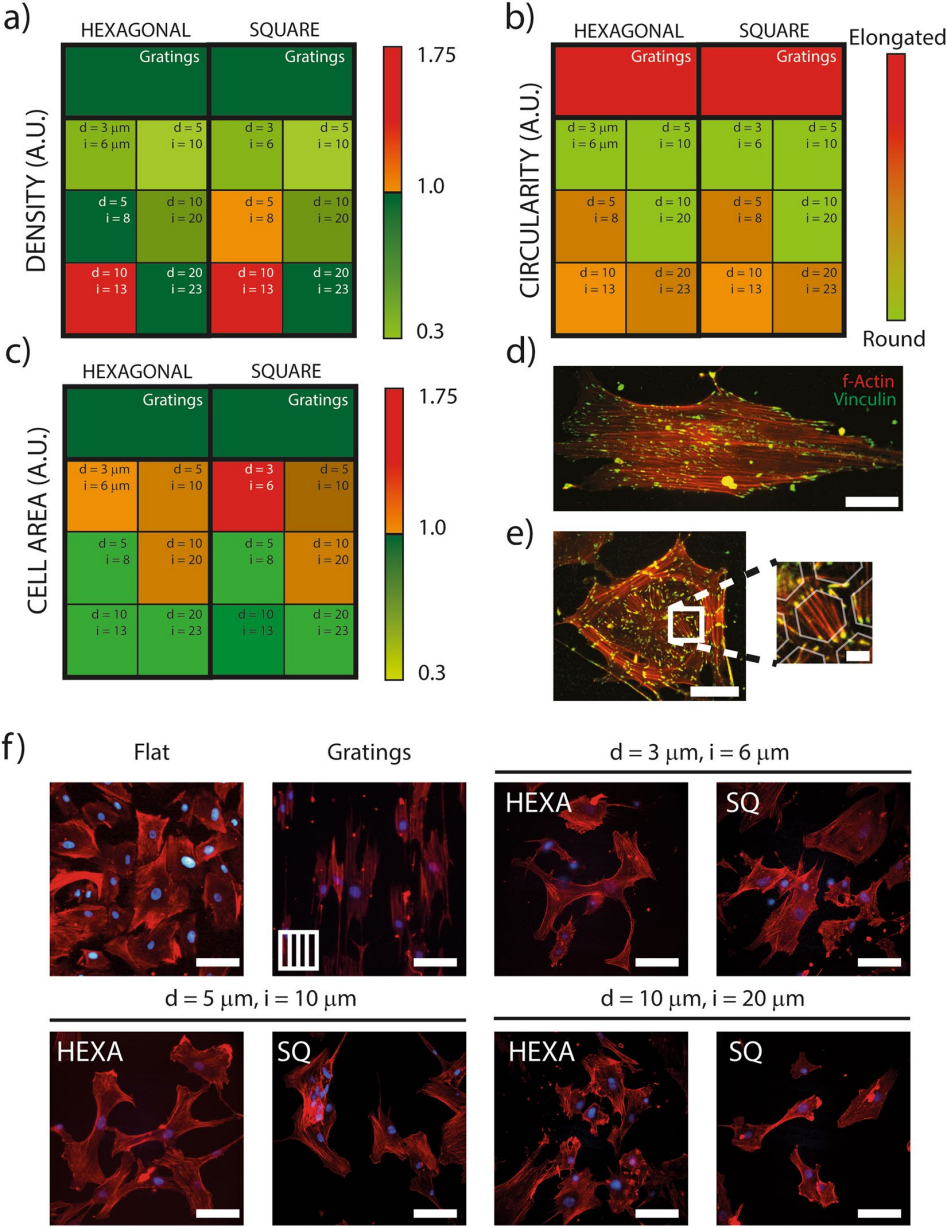
Cell morphology on surface-structured PDMS membranes. HDFs (**a**) density, (**b**) circularity and (**c**) area on different patterns normalized to the HDFs values measured on unstructured flat PDMS surfaces. (**d**) Representative fluorescence image of HDF on flat elastomeric substrate revealing f-Actin (red) and Vinculin (green). Scale bar: 50 µm. (**e**) HDF on Hexa_d20,i23_ PMDS sample. Scale bar: 50 µm. Detail: focal adhesions (green) were preferentially established on the top surface of the walls separating the wells, with short bridging actin fibers (red). The semi-transparent hexagonal structures are artificially overlaid for illustration purposes. Scale bar: 10 µm. (**f**) Representative fluorescence images of HDFs on different patterns revealing F-Actin (red) and nuclei (blue). Scale bar: 80 µm.

The initial screening targeted the adhesion of Human Dermal Fibroblasts (HDFs). *In vivo*, this process enables the deposition of fibrotic tissue at the implant interface and leads to fibrosis^29^. To evaluate the performance of potential anti-adhesive topographies, freshly isolated, primary HDFs from healthy donors were seeded on structured PDMS (Fig. 1). The selected scoring parameters were the cell density and the circularity. The average values were quantified for each experimental condition after 72 h in culture and the results are reported by the graphs in Fig. 2.

The density of adhering cells was significantly reduced on most structured surfaces when compared to the identical flat counterparts. The only exception were surfaces featuring hexagonal or square arrays with d = 10 µm and i = 13 µm which yielded opposite results (Fig. 2a and Supp. Figs 3 and 13). In particular, isotropic and quasi-isotropic topographies curtailed HDF adhesion yielding a 60% reduction. Patterns were evaluated for their anti-adhesive performance, the scalability to cover large surfaces, and the ease of fabrication on both elastomers and biocellulose. Based on these elements hexagonal and square patterns featuring pits with 5 µm diameter were selected for further testing. In particular, these geometries could be generated with high fidelity on the target substrates and yielded a maximal reduction of HDF adhesion (65%, Fig. 2a).

The cell circularity was next considered (Fig. 2b). Circularity significantly increased (i.e. cells were more round) on isotropic and quasi-isotropic patterns (Fig. 2b). Here, a round cell shape indicates that the process of spreading and focal adhesion maturation, leading to the typical spindle-like contour of adhering HDFs, was hindered by the interaction with topography (Fig. 2b). Consistently cells were more elongated on gratings yielding a low circularity in line with previous reports^30–32^.

On anisotropic topographies, the large majority of cells (Supp. Fig. 4, 90%) aligned within 15° to the direction dictated by the gratings. On flat substrates as well as on all other patterned samples (isotropic and quasi-isotropic) no preferential alignment was detected (Supp. Fig. 4).

Taken together, this analysis demonstrates that the majority of tested isotropic and quasi-isotropic patterns of micron-sized pits significantly reduce the adhesion of HDFs to PDMS substrates. (Supp. Fig. 5). In addition, they indicate that this effect is mediated by the inhibition of cell spreading and focal adhesion maturation upon cell interaction with the regular arrays of topographic features.

### Adhesion of HDFs on biocellulose

The analysis of parameters describing the adhesion of HDFs to the topographies under study indicated hexagonal and square patterns featuring pits with 3 μm < *d* < 10 μm and 6 μm < *i* < 20 μm are the ones imparting PDMS surfaces with the most efficient anti-adhesive properties (Fig. 2). To extend the validity of these results to biocellulose, the possibility to transfer the selected geometries by means of guided assisted biolithography (GAB) was next investigated.

**Figure 3:**
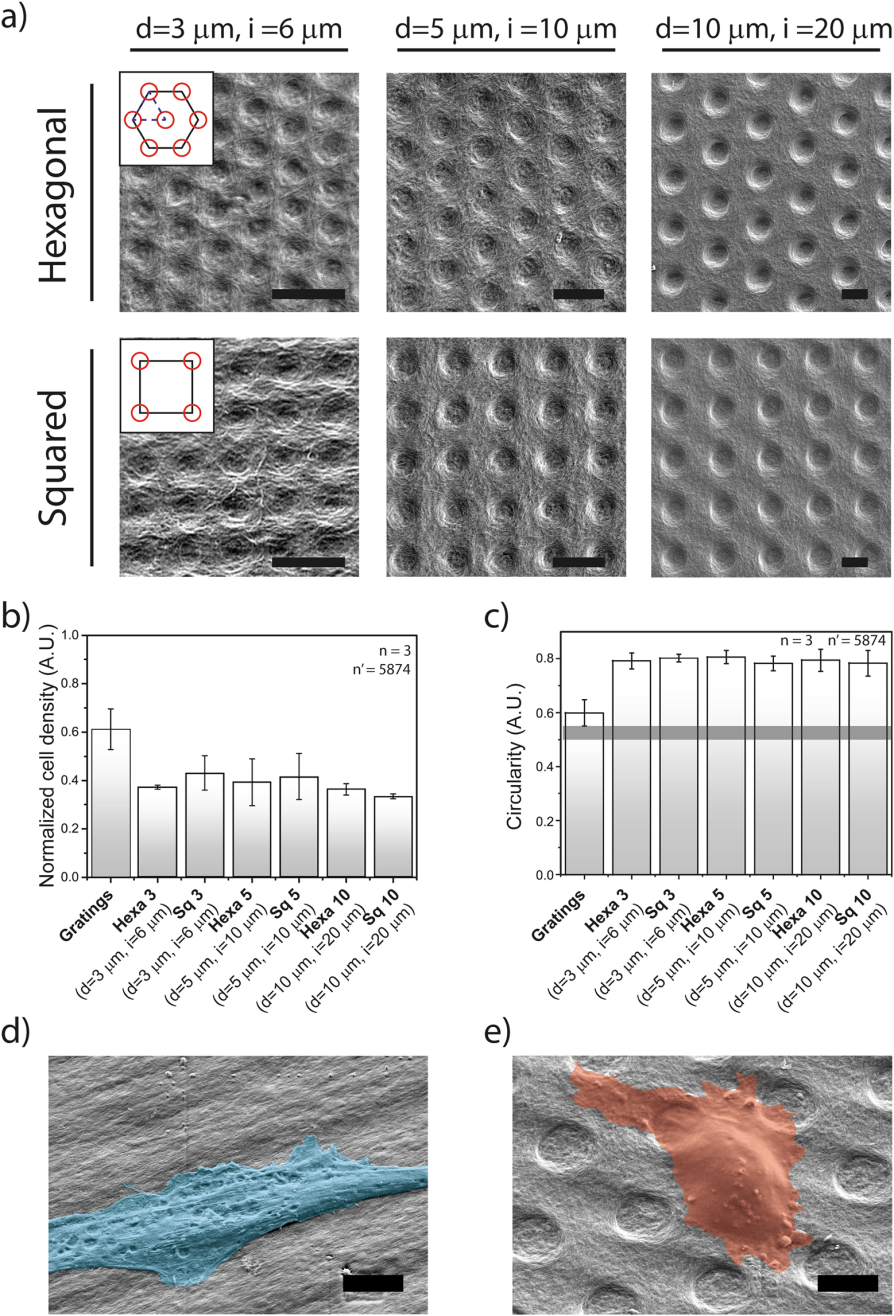
Cell morphology on surface-structured biocellulose. (**a**) First row: hexagonal patterns with well-diameter ranging from 3 µm to 10 µm. Second row: squared patterns with microwells diameter ranging from 3 µm to 10 µm. Scale bar: 10 µm. (**b**) HDFs density on different patterns on biocellulose normalized to the HDFs density measured on unstructured biocellulose substrates. (**c**) HDFs circularity on different patterns. Representative scanning electron microscopy images of HDFs on (**d**) gratings and on (**e**) Hexa10 (d = 10 µm, i = 20 µm) micropatterns. Scale bar: 10 µm. Cell surface is colored for visualization purposes.

In GAB, structured PDMS substrates are used as molds to yield a negative replica of featured surface geometries^17^. In particular, the vertical feature size of the silicone molds was calibrated to obtain elements with the desired pit depth (Methods). Biocellulose substrates manufactured by GAB presented the typical matrix-like structure, with intertwined and closely packed nano-ribbons shaping the imprints (Fig. 3). The SEM micrographs in Fig. 3a, render the quality of the transferred patterns. The fidelity of the GAB replica-molding process had a deviation from the nominal values ranging between 0.2% and 10% (Table 1), as previously reported^17^. In these tests 3 isotropic and 3 quasi-isotropic pit geometries were included. Here, the adhesion of HDFs was initially evaluated in comparison with identical flat substrates and gratings (Fig. 3 and Supp. Figs 6 and 7).

**Table 1:** Fidelity of guided assembly bio-lithographic process.

Topography	Initial		Biocellulose Replica		Lateral Size Replica Fidelity (%)	
	*d*_*mold*_ (µm)	*i*_*mold*_ (µm)	*d*_*cell*_ (µm)	*i*_*cell*_ (µm)	*d*	*i*
Square 3	3	6	3.15 ± 0.21	5.50 ± 0.37	5.13%	8.34%
Hexa 3			2.73 ± 0.20	5.85 ± 0.35	8.91%	2.51%
Square 5	5	10	4.97 ± 0.14	9.54 ± 0.30	0.57%	4.55%
Hexa 5			5.18 ± 0.13	9.71 ± 0.25	3.64%	2.91%
Square 10	10	20	10.02 ± 0.33	19.01 ± 0.58	0.17%	4.93%
Hexa 10			9.91 ± 0.38	19.50 ± 0.48	0.92%	2.49%

The initial design columns report the planned size of the microstructures, while the biocellulose replica columns report the respective measured values. The replica fidelity calculation gives an estimate of the relative deviation of the bio-lithographic replica with respect to the theoretical design.

Cell density and circularity were used as descriptors of HDFs adhesion to biocellulose upon seeding and incubation for 72 h. The results obtained reproduced those reported on analogous PDMS samples. In particular, all patterns reduced cell density as compared to the flat control, with maximal reduction of 65% (Fig. 3b, Hexa-3, Hexa-10, Sq-10). The cell circularity was consistently increased with cells appearing more round on all isotropic and quasi-isotropic geometries (Fig. 3c, d, e and Supp. Figs 6 and 7). Interestingly, gratings on biocellulose did not support cell elongation to the extent measured on PDMS, thus indicating an overall reduced interaction with the material. Altogether, these results confirm that the anti-adhesive properties of selected isotropic and quasi-isotropic patterns of pits, are independent of the bulk material upon which they are implemented.

### Proliferation and differentiation of HDFs on anti-adhesive topographies

To gain in-depth information on cell differentiation and colonization of the substrate, the long-term fate of HDFs interacting with biocellulose was next investigated. After 1 week of cell culture, the initial reduction of cell density detected on isotropic and quasi-isotropic patterns further increased to 75% as compared to the flat control (Supp. Fig. 8). HDFs require the establishment of mature focal adhesions to the substrate in order to proceed in the cell cycle^33^ therefore the measured effect could be related to a reduced proliferation rate on anti-adhesive topographies. The selective incorporation and subsequent fluorescent labeling of EdU in the DNA of proliferating cell provides an easy quantification of cell division. This data demonstrates that the long-term interaction with anti-adhesive topographies significantly hinders cell proliferation (of 90%), thus establishing a link between the inhibition of adhesion and spreading, and the arrest of the cell cycle (Supp. Figs 9 and 10).

Adhesion and proliferation of HDFs at the interface with biomaterials are necessary prerequisites for the onset of fibrosis. However, they are alone not sufficient to execute the complex ensuing phases of fibrotic matrix deposition and contraction. These specialized activities require the differentiation of fibroblasts into contractile myofibroblasts, a process that is activated by the interaction with foreign materials. To detect myofibroblasts differentiation upon contact with the substrates under investigation, the expression of a well-established reporter (α-Smooth Muscle Actin, α-SMA) was revealed^34,35^. In addition, the differentiation of HDFs contacting tissue culture polystyrene (TCPS) and silicones (MED 6015) was evaluated as a positive (i.e. pro-fibrotic) control. On these substrates, the labeling of α-SMA rendered an intense signal from well-resolvable actin stress fibers, the all-marks of contractile myofibroblasts (Supp. Fig. 11)^35^.

Interaction between HDFs and biocellulose yielded low levels of myofibroblast differentiation indicating that the bulk material properties do not support the activation of the required signals. Importantly, the implementation of anti-adhesive topographies introduced a significant additional inhibition of differentiation, as indicated by the consistent reduction of α-SMA expression (Supp. Fig. 11).

Altogether, these results establish the anti-adhesive topographies as effective inhibitors of fibroblast proliferation and differentiation into contractile cells, thus further supporting their role in the prevention of fibrosis.

### Macrophage interaction with anti-adhesive topographies

The signals leading to fibrosis are initiated upon the early adhesion of inflammatory precursors to the surface of implanted biomaterials. Their subsequent differentiation into pro-inflammatory cells^36^ requires the expression of a set of genes which drive their differentiation and sustain the secretion of chemokines altogether enabling the inflammatory process. In this complex scenario a pivotal role is played by macrophages which differentiate from monocytes upon neutrophil signaling^1^.

The next set of experiments was therefore aimed at evaluating the performance of the anti-adhesive topographies in reducing the adhesion and activation of macrophages. THP-1 cells, a well-established cell line for the investigation of human macrophage activity^37^ were used for these experiments, and their adhesion and differentiation was compared upon interaction with TCPS, silicones, and biocellulose substrates.

**Figure 4:**
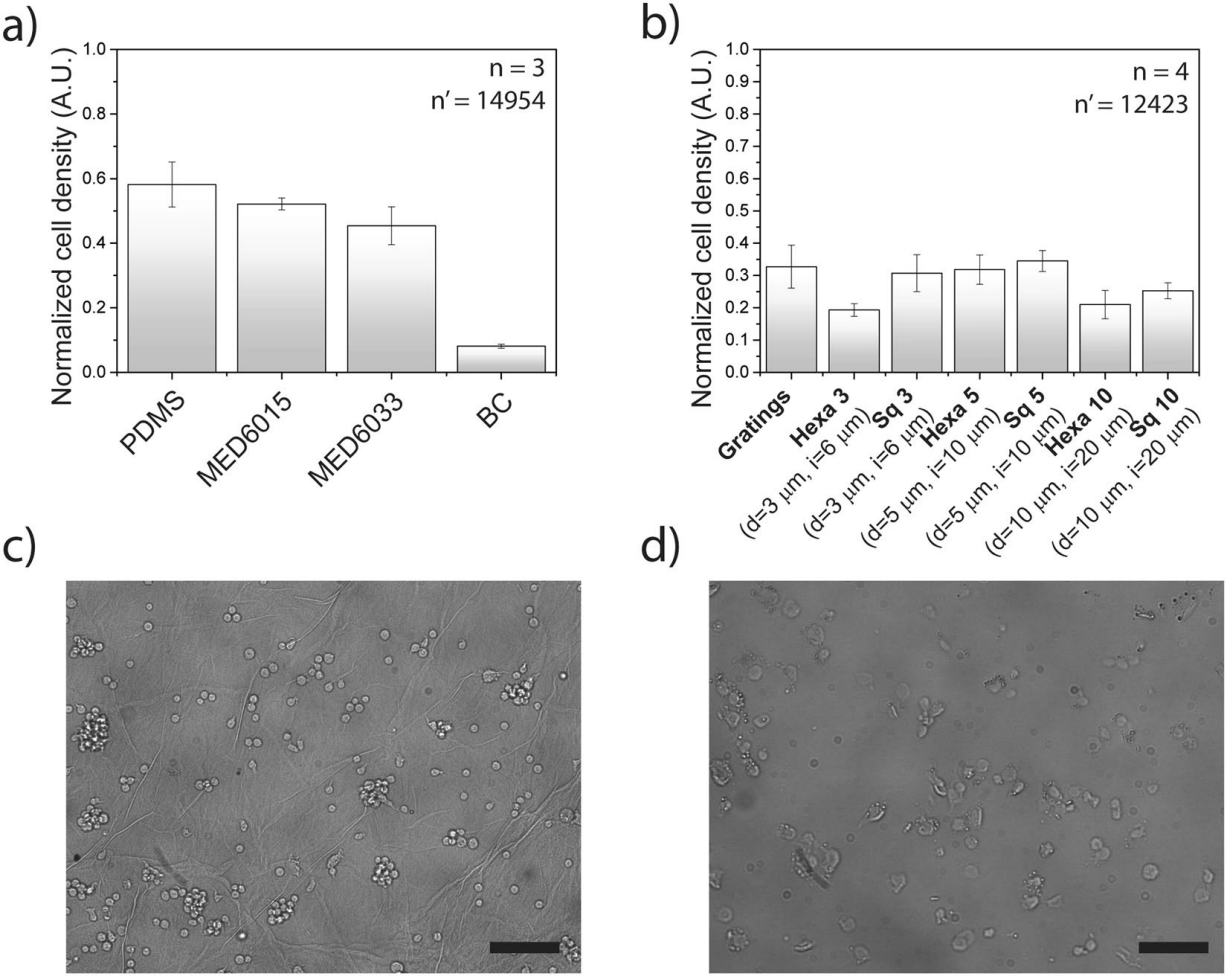
Macrophages adhesion onto different materials. (**a**) THP-1 cells density on different materials normalized to the THP-1 cells density measured on Tissue Culture Plastic. (**b**) THP-1 cells density on different patterns on biocellulose normalized to the THP-1 cells density measured on unstructured biocellulose substrates. Representative widefield images of THP-1 macrophages on (**c**) microstructured biocellulose and on (**d**) MED 6015. Scale bar: 100 µm.

THP-1 cells adhered well to the surface of TCPS, which was selected as the positive control. Adhesion to silicones (including PDMS, MED 6015 and MED 6033), was relatively less efficient, showing a 40–60% reduction of resulting cell density (Fig. 4a). The most dramatic effect was however detected on biocellulose, on which the cells density was reduced of 90% or more. Altogether, this data demonstrates that macrophage adhesion varies greatly as a function of the presented material interface and establish biocellulose as a low affinity substrate.

To decouple the effect of anti-adhesive topographies, a direct comparison was performed between flat biocellulose and corresponding substrates structured with the selected isotropic and quasi-isotropic pit patterns (Fig. 4b). The presence of anti-adhesive topographies inhibited cell adhesion yielding a further average density reduction of 75% as compared to flat biocellulose. The best performing pattern featured a hexagonal pit array with *d* = 3 μm and *i* = 3 μm and yielded a cell density reduction of 82%.

Altogether these results demonstrate that the bulk material composition and its surface geometry independently cooperate to define the substrate affinity to the adhesion of macrophages.

**Figure 5:**
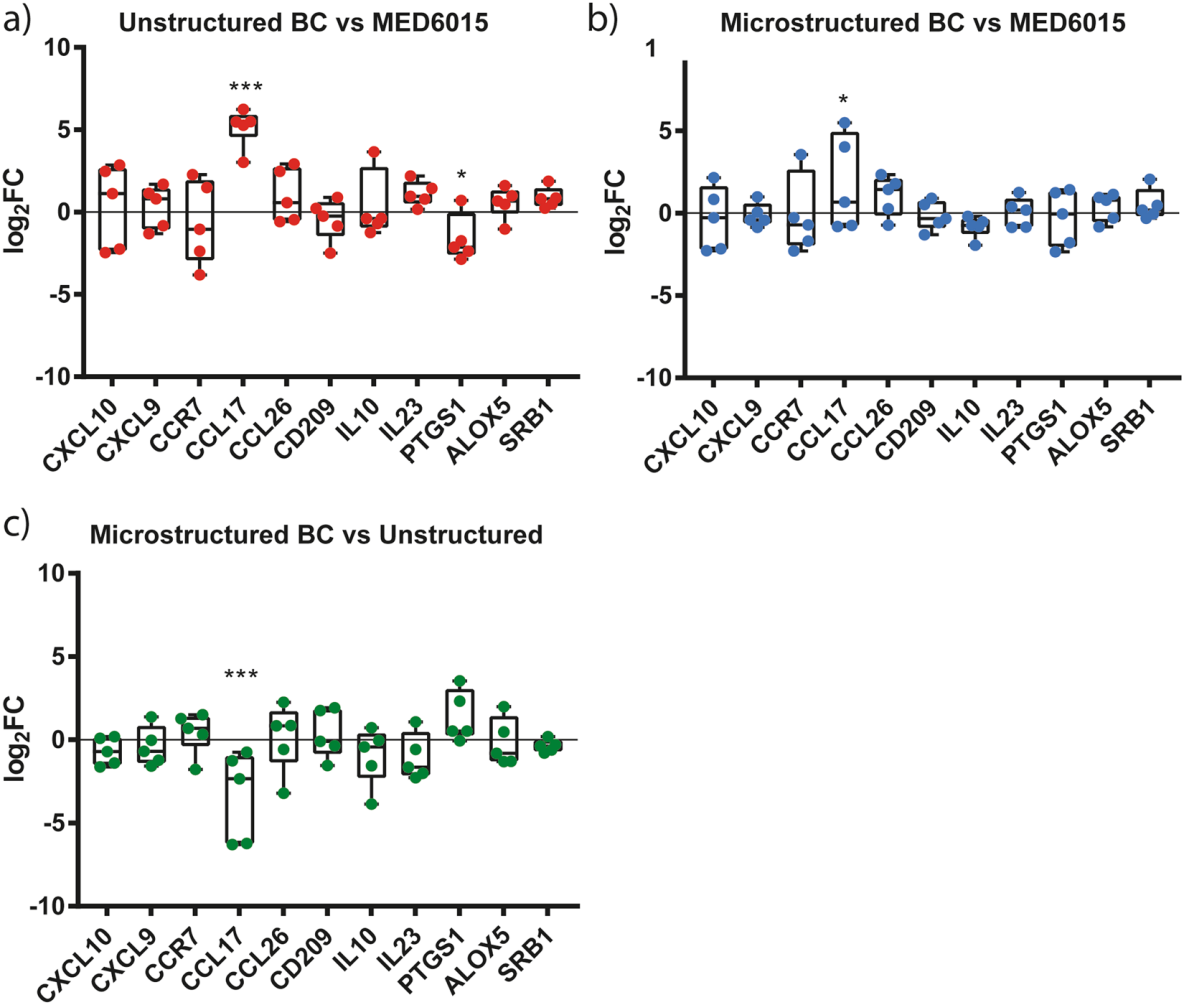
Expression levels of selected pro- and anti-inflammatory genes. The expression levels of selected target genes were calculated in fold change (2-ddCt) and plotted in the graphs (antilog scale). n = 5 were used for the analysis. (**a**,**b**) The additional x axis at y = 0 indicates the expression levels of each specific marker in THP-1 cells cultured onto silicone coated plates. CCL17 is significantly up-regulated by cells seeded on the flat surface (p < 0.001) and on the microstructured (Hexa5, d = 5 µm, i = 10 µm) cellulose (p = 0.02) with comparison to the expression levels of the same markers onto silicone. PTGS1 appears to be significantly down regulated (p = 0.03) in THP-1 cells cultured onto flat surfaces. (**c**) The x axis at y = 0 indicates the expression levels of each marker in THP-1 cells cultured onto flat surfaces. CCL17 appears down-regulated (p < 0.001) microstructured (Hexa5, d = 5 µm, i = 10 µm) cellulose with comparison to unstructured cellulose.

Finally, to determine whether the interaction with the substrates under investigation had a direct effect on the gene expression pattern of THP-1 cells, a qPCR analysis was performed. The lysate of cells adhering to silicones or biocellulose was processed for RNA extraction and quantitative gene expression comparison. The relative expression of a panel of genes defining the pro-inflammatory activation of macrophages is reported in Fig. 5.

The only significant difference in the expression of the selected genes was detected for CCL17, which sustains the secretion of a chemokine known to attract immunitary T-cells^38^. Expression of the CCL17 gene was significantly increased upon interaction with biocellulose, however the presence of anti-adhesive topography caused downregulation with respect to the unstructured biocellulose. In particular, the CCL17/TARC protein promotes the migration of T cells, through the specific binding to the CCR4 receptor^39^. Its overexpression is therefore linked to an increased immunological activity and is normally encountered as part of the immune response to local infections. In the specific case reported by our *in vitro* tests, the induction of CCL17 points to a higher LPS content in biocellulose samples, as compared to other substrates. This parameter (i.e. the LPS residual content) can vary greatly between biocellulose substrates, due to a number of factors affecting the cleaning step. We cannot therefore exclude that this specific results (although minor) may be the signal of residual LPS contamination in our probes. This however, shall not be ascribed to biocellulose per se. Taken together, these results indicate that the interaction with surface-microstructured biocellulose does not cause pro-inflammatory activation of macrophages. A distinctive effect of anti-adhesive topography could not be resolved by the analysis performed.

### Degradability of the material

Biocellulose substrates featuring anti-adhesive geometries were fabricated for *in vivo* testing of their anti-fibrotic effect^40^. For this investigation, the GAB molding procedure was up-scaled yielding large biocellulose foils of 200 cm^2^, which presented the selected hexagonal pit array (Hexa10) on the entire external surface. For these tests the surface pattern Hexa10 was selected based on its good performance in the *in vitro* tests (Fig. 2 and Supp. Fig. 13) and the optimal compatibility with the GAB molding process on large substrates yielding a reliable transfer of topographic features^17^. The biocellulose layer was implanted in a surgical pocket in direct contact with interstitial tissues of adult pigs. The results and details of this pre-clinical investigation are reported in a separate dedicated publication^40^.

The implanted materials were retrieved after 6 weeks and the degradation of biocellulose was evaluated. Particular attention was paid to the deterioration of the imprinted topographic features, which provides a direct indication on the durability of their anti-adhesive effect. The SEM micrographs of explanted biocellulose substrates (Supp. Fig. 12) show that the isotropic array was largely retained *in vivo* therefore supporting its application for the protection of implantable biomaterials.

## Conclusion

The physiological regeneration of the tissues surrounding an implant is critical to ensure its long-term integration in the body. To this end, protective strategies for the management of the surgical pocket aim at avoiding the onset of adverse inflammatory events, which over the course of the device lifetime can cause an early revision or render a dangerous device exchange procedure.

Most of the biological processes leading to healthy tissue healing or to fibrosis, depend on the early interaction taking place at the interface between the implant and the recipient cells and tissues. If the synthetic surface is recognized as a foreign body, the adhesion and activation of inflammatory cells trigger a diverging reaction tending towards the formation of fibrotic tissue and its ensuing contraction.

Much of the implant fate is therefore decided in these initial phases. Soft silicone implants are doomed to lose their aesthetic function when the stiffening caused by capsular contracture causes deformation, discomfort, and pain. Non-deformable implants do not suffer from such failure. However, the early misrecognition leads to the accumulation of a dense fibrotic tissue, which is dangerous during the device exchange, requiring a delicate procedure of excision and extraction.

Anti-fibrotic strategies must therefore act at the level of the surgical pocket and hinder the inflammatory response leading to foreign body reaction. In short, they must hide the implant from the body. The solution proposed here is based on rendering the implant non-adhesive to the cells which initiate the signaling and actuate the process of fibrosis. Our data demonstrate that this is made possible by implementing an optimized surface geometry. The anti-adhesive geometry is a symmetric array of microstructured topographical features representing physical obstacles to the establishment and maturation of cell adhesion. The spreading of pro-inflammatory cells (i.e. macrophages) and of their effectors (i.e. fibroblasts) are necessary prerequisites for their activation toward fibrosis. Therefore, the inhibition of the initial biological recognition interrupts the signaling cascade avoiding the formation of fibrotic tissue.

The benefit of an anti-adhesive topography is threefold. First, it can be implemented on several target biomaterials, including silicones, thermoplastic polymers, and hydrogels, by means of well-established lithographic protocols. This surface functionalization is limited to the external surface, and thus does not modify the implant architecture and function. The structure upscaling to large surfaces is easy and can fit the size of current implants. Second, our *in vitro* experiments show that the anti-adhesive effect of topography is independent from other biochemical properties of the biomaterial on which is implemented. Thus, it provides a significant additive level of protection, which can be implemented directly on the device or on the external surface of a protective biocellulose layer. Finally, the pristine geometry imposed during fabrication is not altered by the coating with matrix proteins. The *in vivo* exposure to living animal tissues does not degrade the surface configuration supporting the long-term anti-adhesive effect of the surface microstructure.

Altogether, we have established a novel strategy to protect artificial implants from foreign body reaction and its complications. It consists of the implementation of a continuous symmetric array of topographic microfeatures, which imparts the surface of a target biomaterial with anti-adhesive properties, hampering the activation of macrophages and fibroblasts. We envision the specific application of this strategy in the protection of silicone implants from capsular contracture or in its general implementation to an anti-fibrotic layer comprised of well-tolerated biomaterials such as biocellulose.

## Methods

### PDMS Mold Fabrication

Microstructured molds were manufactured through standard soft lithography^41^ with polydimethylsiloxane (PDMS, Dow Corning, USA) at a 1:10 mixing ratio. Briefly, the mixture of PDMS monomeric solution and curing agent was degassed in a vacuum chamber for 10 min to remove trapped air and promptly poured onto a silicon wafer presenting the micropattern of interest. Master molds were produced with a double layer photolithographic process on p-type doped silicon wafers. A first 1500 nm layer of negative resist (AZ2020, Microchemicals GmbH) was applied onto the wafer through spin coating and flood exposed to UV light (365 nm) to fully crosslink the resist and subsequently developed to remove uncross-linked residues. A second layer of negative resist (AZ2020, Microchemicals GmbH) or positive resist (AZ 6632, Microchemicals GmbH) was applied and patterned through UV-lithography in order to perform soft-lithography for cell adhesion experiments, or Guided Assembly based Bio-lithography (GAB), respectively. Several types of patterns were manufactured, which featured well diameters ranging from 3 to 20 µm and well center-to-center distances ranging from 6 to 23 µm (Table 1 and Supp. Table 1). The well depth was 1.4 or 3.3 µm.

The selected dimensions were identified within a parametric space, which is limited by several factors:

1. Geometric constraints: the shape of the elemental cell must be preserved unchanged, meaning that the distance between the centers *i* of the wells cannot be smaller than the diameter *d* of the well itself.
2. Fabrication constraints:
  - The diameter of the wells cannot be smaller than the resolution limit of the photolithographic process itself (1 µm).
  - The aspect ratio of the “walls” separating the wells has been limited to a value of 0.5 (and therefore to an absolute value of 3 µm). The reason for this constraint relies in the reproducibility and yield of the soft lithographic process and in the necessity to rule out any possible deformation of the substrate from the analysis.

Templates featuring 3.3 µm deep micro-pillars, were used in production of microstructured biocellulose substrates with GAB, while a second and a third batch of molds, featuring 1.4 µm deep micro-wells, were used directly in *in vitro* experiments. Two different sets of molds were used to account for the limited resolution of the GAB process to transfer surface topography in the vertical direction.

After pouring the PDMS solution on the master molds, it was briefly degassed for a second time and cured for 4 h at 60 °C^42^. The cured PDMS patches were then carefully separated from the master mold using tweezers. The integrity and the characteristic dimensions of the patterns were then validated using an optical, non-destructive, interferometry based surface profilemeter (White Light Interferometer, Zygo, USA).

### Guided Assembly-based Biolithography (GAB)

Wild type *Acetobacter Xylinum* strain ATCC-700178 (LGC Standards, Wesel, Germany) was used for biocellulose fermentation. The bacteria were grown in a fully synthetic medium^43^ sterilized by autoclaving. In order to generate surface-structured biocellulose substrates, 1 mL of medium was distributed in to standard 24-well-plates. Microstructured PDMS molds were then placed in each well, with surface topography facing the liquid. Bacterial cultures were incubated at 26.5 °C in saturated humidity and steady environments for 7 days. At the end of the culturing period, PDMS molds were removed, surface microstructured cellulose substrates (GAB substrates) were collected and washed in NaOH 1 M for 8 h at 80 °C, and subsequently in de-ionized (DI) water at RT until neutral pH was reestablished. The GAB substrates were purified from endotoxin-rich residues from the bacteria cell wall (LPS) with at least 3 washings of 2 h in sterile, pyrogen-free, endotoxin-free water for injections (*Aqua ad Iniectabilia*, Braun AG, Germany). The substrates were then dehydrated at room temperature and stored in dry state for up to 1 month. Before each cell culture experiment, dehydrated cellulose substrates were then rehydrated with DI water. Unless otherwise specified, all reported experiments were performed using re-hydrated substrates.

### Mammalian Cell cultures

Human dermal foreskin fibroblasts (HDF) were supplied by the Tissue Biology Research Unit (Department of Surgery, University Children’s Hospital Zurich, CH) and obtained according to the principles of the Declaration of Helsinki. Briefly, the cells were cultured in DMEM-1640 medium supplemented with 10% v/v fetal bovine serum (FBS), 2 mML-glutamine, 100 U ml^−1^ penicillin and 100 mg ml^−1^ streptomycin (all from Sigma Aldrich) and maintained at 37 °C and 5% CO_2_. In all reported experiments, cells with less than ten passages *in vitro* were used.

THP-1 cells were cultivated in RPMI-1640 medium (Sigma Aldrich), supplemented with 10% v/v fetal bovine serum (FBS), 2 Mm l-glutamine, 100 U mL^−1^ penicillin and 100 mg mL^−1^ streptomycin (all Sigma Aldrich) and maintained at 37 °C with 5% CO_2_. They were split every 2 or 3 days, in order to maintain their concentration around 2 × 10^5^ cells/ml.

Non-coated, pristine PDMS and bio-synthesized cellulose substrates were not suitable for HDFs adhesion and growth; therefore, to independently assess the effect of the different patterns, sterile substrates were coated with fibronectin before cell seeding. Briefly, the PDMS molds were treated with oxygen plasma (0.7 mbar, 30 s, 60 W) to increase hydrophilicity and immediately incubated in 1 µg/ml Fibronectin (Sigma-Aldrich, USA) in 1X PBS for 1 h at 37 C. Subsequently, the samples were rinsed three times in PBS to remove excess of fibronectin and then incubated at 37 C in growth medium prior to cell seeding. Biocellulose substrates underwent a similar coating, but with an extended incubation time of 8 h in the fibronectin solution. Non-confluent HDFs were then trypsinized and seeded on the substrates at a density of 10^4^ cells/cm^−2^. Cells were incubated for 72 h before fixation and imaging.

For the experiments to evaluate macrophage adhesion on different materials, a concentration of 7 × 10^4^ cells/cm^2^ was seeded on uncoated substrates^44^, in 24-well plates with 1 ml of X-Vivo 15 serum-free medium (Lonza) and 10 ng/ml of Phorbol 12-myristate 13-acetate (PMA, Sigma Aldrich), which is close to the smallest concentration proved to be needed in order to stimulate monocyte differentiation into a macrophage-like phenotype without inducing up-regulation of undesired genes^45^. After seeding, cells were incubated for 72 h before fixation and imaging.

On the other hand, experiments performed for gene analysis required approximately 10^6^ of cells as output. They were performed in petri dishes of 10 cm of diameter each, with a seeding concentration of between 2.5 × 10^5^ and 3.2 × 10^5^ cells/ml, 30 ml of X-Vivo 15 and 10 ng/ml of PMA. After 72 h of incubation, cells were detached from the surface using 0.25% Trypsin-EDTA (Fisher Thermo Scientific) at 37 °C for 5 min, collected in centrifuge tubes and immediately frozen at −80 °C. Only cells cultivated for less than 25 passages were used for experiments.

### Immunostaining

To visualize the focal adhesions established by cells on the biocellulose substrates the following primary antibody was used: mouse anti-vinculin (V4505) from Sigma. The secondary antibody was a donkey anti-mouse-alexa-488 (A-21202) from Invitrogen. Filamentous actin was visualized using TRITC-phalloidin (Sigma).

Cells were fixed for 20 min with 2% paraformaldehyde (PFA) in PBS at room temperature (RT). The cells were then permeabilized with 1% Triton-X100 in PBS for 5 min. After washing the samples three times for 5 min with PBS, they were incubated with 5% bovine serum albumin (BSA) in PBS for 1 h at RT to block non-specific antibody binding. The samples were incubated with TRITC-phalloidin (Sigma, USA) and with mouse anti-vinculin primary antibody overnight at 4 °C. Subsequently, the samples were rinsed four times for 1 h with 5% BSA in PBS and then incubated with donkey anti-mouse-alexa-488 secondary antibody for 45 min at RT. Finally, the samples were washed three times (1 h each) in PBS, then washed with 0.1% Hoechst in PBS for 15 minutes and immediately imaged.

### Wide-field Microscopy

Wide-field imaging was performed with a 20X, 0.70 NA long-distance objective (Plan Fluor, Nikon) using an inverted Nikon-Ti wide-field microscope (Nikon, Japan) equipped with an Orca R-2 CCD camera (Hamamatsu Photonics, Japan).

Confocal images of cells were collected using a 40X, 1.3 NA, oil immersion objective (Plan-Apo, Nikon) with a Nikon-Ti Eclipse spinning disk confocal microscope (Nikon, Japan) equipped with an iXon Ultra 888 EMCCD (Andor, UK).

After sputtering of approximately 5 nm of Gold/Palladium on the surface, samples were imaged using a scanning electron microscope (Hitachi High Technologies Europe, Germany) with detection of signal from secondary electrons.

### Image Analysis

Confocal Z-stacks were projected using the maximum intensity projection function of ImageJ (National Institutes of Health, USA). Cell profiles were then manually drawn and the cell area, orientation and circularity were measured using the fit ellipse tool of ImageJ. Specifically, the cell area was obtained from the actin staining using the ‘freehand tool’ of ImageJ. To render a reliable measure of cell density, the process of seeding was carefully adjusted to ensure a uniform distribution of cells on the substrate under analysis. The initial seeding procedure was performed starting from a large volume of cell suspension and avoiding the formation of cell clusters. The endpoint analysis was performed following a standard sampling approach, randomly choosing a fixed number of 10 fields of view. The analysis was then obtained using the ‘analyze particles’ tool of ImageJ on the nuclear DAPI channel. Cell orientation was defined as the angle of the major axis of the fitted ellipse with respect to the principal direction of the pattern. The range of possible alignment was between 0 and 90°. The cell density on flat and structured cellulose was measured using a custom algorithm based on the Analyze Particles tool of ImageJ (National Institutes of Health, USA). The total number of cell nuclei detected in each field of view was divided by the relative surface.

### RNA extraction and reverse transcription

Total RNA was extracted using the GenElute Mammalian Total RNA Kit (Sigma), following the manufacturer’s instructions. Reverse transcription was performed for each sample in 20 µl reaction mixture containing 1 µg of RNA, 1× PCR buffer, 5 mM MgCl_2_, 10 mM of each dNTP, 0.625 µM oligo d(T)_16_, 1.875 µM random hexamers, 20 U RNase inhibitor and 50 U MuLV reverse transcriptase (all from Life Technologies). The conditions for the reverse transcription were the following: 25 °C for 10 min, 42 °C for 1 h, followed by 99 °C for 5 min.

### Quantitative Real-Time PCR

The resulting cDNA was amplified in duplicate by Quantitative Real-Time PCR in 10 µl reaction mixture with 200 nM of each specific primer (Table 2) and 1× Fast Syber Green qPCR MasterMix (Life Technologies). For the amplification reaction, StudioQuant 7 from Applied Biosystem was used. The amplification program was set as follows: 95 °C for 5 min, followed by 40 cycles at 95 °C for 10 sec, 60 °C for 15 sec, 72 °C for 20 sec. GAPDH and 18S served as housekeeping genes and their amplification data were averaged and used for sample normalization. Excel Software was used for the comparative quantification analysis.

**Table 2:** List of primers (forward and reverse, respectively) used for real time PCR analysis.

*Gene name*	*Primers*
*CXCL10*	5′ GCA AGC CAA TTT TGT CCA CG 3′ 5′ ACA TTT CCT TGC TAA CTG CTT TCA G 3′
*CXCL9*	5′ GAC CTT AAA CAA TTT GCC CCA AG 3′ 5′ TCC TTC ACC CCC ATC TGC TGA ATC TGG 3′
*CCR7*	5′ GAA AGT CCA GAA ACT GTT CCC ACC TGC 3′ 5′ CCC CTC TGA AGA ACC GAA CCA CTC CTT 3′
*CCL17*	5′ CCA GGG ATG CCA TCG TTT TTG TAA CTG TGC 3′ 5′ CCT CAC TGT GGC TCT TCT TCG TCC CTG GAA 3′
*CCL26*	5′ GCC TGA TTT GCA GCA TCA TGA TGG 3′ 5′ CGG ATG ACA ATT CAG CTG AGT CAC 3′
*DC-SIGN*	5′ TCG AGG ATA CAA GAG CTT AGC A 3′ 5′ AAG GAG CCC AGC CAA GAG 3′
*IL10*	5′ CTG TGA AAA CAA GAG CAA GGC 3′ 5′ GAA GCT TCT GTT GGC TCC C 3′
*IL23*	5′ GCA GAT TCC AAG CCT CAG TC 3′ 5′ TTC AAC ATA TGC AGG TCC CA 3′
*PTGS1*	5′ CGC CAG TGA ATC CCT GTT GTT 3′ 5′ AAG GTG GCA TTG ACA AAC TCC 3′
*ALOX5*	5′ CGC CGA CTT TGA GAA AAT CT 3′ 5′ GGC TGC ACT CTA CCA TCT CC 3′
*SRB1*	5′ TCC TCA CTT CCT CAA CGC TG 3′ 5′ TCC CAG TTT GTC CAA TGC C 3′
*GAPDH*	5′ GTC AGT GGT GGA CCT GAC CT 3′ 5′ ACC TGG TGC TCA GTG TAG CC 3′
*18S*	5′ CCC GGG GAG GTA GTG ACG AAA AAT 3′ 5′ GCC CGC TCC CAA GAT CCA ACT AC 3′

### Animal experiment

During a pre-clinical study on facilitating easy Cardiovascular Implantable Electronic Devices (CIEDs) exchanges and redo-procedures on the device pocket, large size biocellulose membranes were used to wrap commercial pacemakers (PMs) before implantation in a pig model. In brief, the membranes were fitted around the devices and sutured in order to generate a conformal cloaking of the pacemakers and the proximal leads^40^. For the purpose of this study, only the overall integrity and state of the surface were investigated with optical and electron microscopy for signs of degradation or damages. The study was performed in the framework of Article 18 Animal Welfare Act (TSchG), article 141 Animal Welfare Ordinance (TSchV), article 30 Animal Experimentation Ordinance (TVV) after permission granted by license 162/2014 issued by the Kantonales Veterinäramt, Zürich. The used protocols followed the Standard Operating Procedures of the Animal Facility (BZL) of the University Hospital of Zürich, in accordance with the ARRIVE Guidelines.

### Statistical analysis

For all reported tests three independent experiments were performed and mean values (with n = 3**)** were calculated. All quantitative measurements reported are expressed as mean values ± the standard error of the mean. The total number of cells involved in each of the presented analysis is indicated in the graphs. For the gene expression analysis, statistical analysis was performed using Graphpad Prism. The normal distribution of the amplification data acquired was verified with D’Agostino-Pearson, Shapiro-Wilk and KS normality test. Repeated Measures Two-way ANOVA test with Sidak correction for multiple comparison was applied to the untransformed dCt data to investigate significant inter sample differences in gene expression levels.

## Electronic supplementary material

Supplementary Information

Supplementary Video 1
