# Supplementary material for: A micron-scale surface topography design reducing cell adhesion to implanted materials
Source: Sci Rep. 2018 Jul 18;8:10887. doi: 10.1038/s41598-018-29167-2 (PMC6052105; doi:10.1038/s41598-018-29167-2)
Supplement: Supplementary file 1 — Supplementary Information [file 41598_2018_29167_MOESM1_ESM.pdf]

A micron-scale surface topography design reducing cell adhesion to implanted materials

Francesco Robotti[a], *Simone Bottan*[b], *Federica Fraschetti*[a], *Anna Mallone*[c], *Giovanni Pellegrini*[d], *Nicole Lindenblatt*[e], *Christoph Starck*[f], *Volkmar Falk*[f], *Dimos Poulikakos*[a] *and Aldo Ferrari*[a]*.

a. Laboratory of Thermodynamics in Emerging Technologies, Department of Mechanical and Process Engineering, ETH Zurich, Sonneggstrasse 3, CH-8092 Zurich, Switzerland
b. Wyss Zurich, ETH Zurich/University of Zurich, Zurich, Switzerland
c. Institute for Regenerative Medicine, University of Zurich, Zurich, Switzerland
d. Laboratory for Animal Model Pathology, Institute of Veterinary Pathology, Vetsuisse Faculty, University of Zurich, Zurich, Switzerland
e. Department of Plastic and Hand Surgery, University Hospital Zurich, Zurich, Switzerland
f. Department of Cardiothoracic and Vascular Surgery, German Heart Institute Berlin, Berlin, Germany

Keywords:

* To whom correspondence should be addressed.

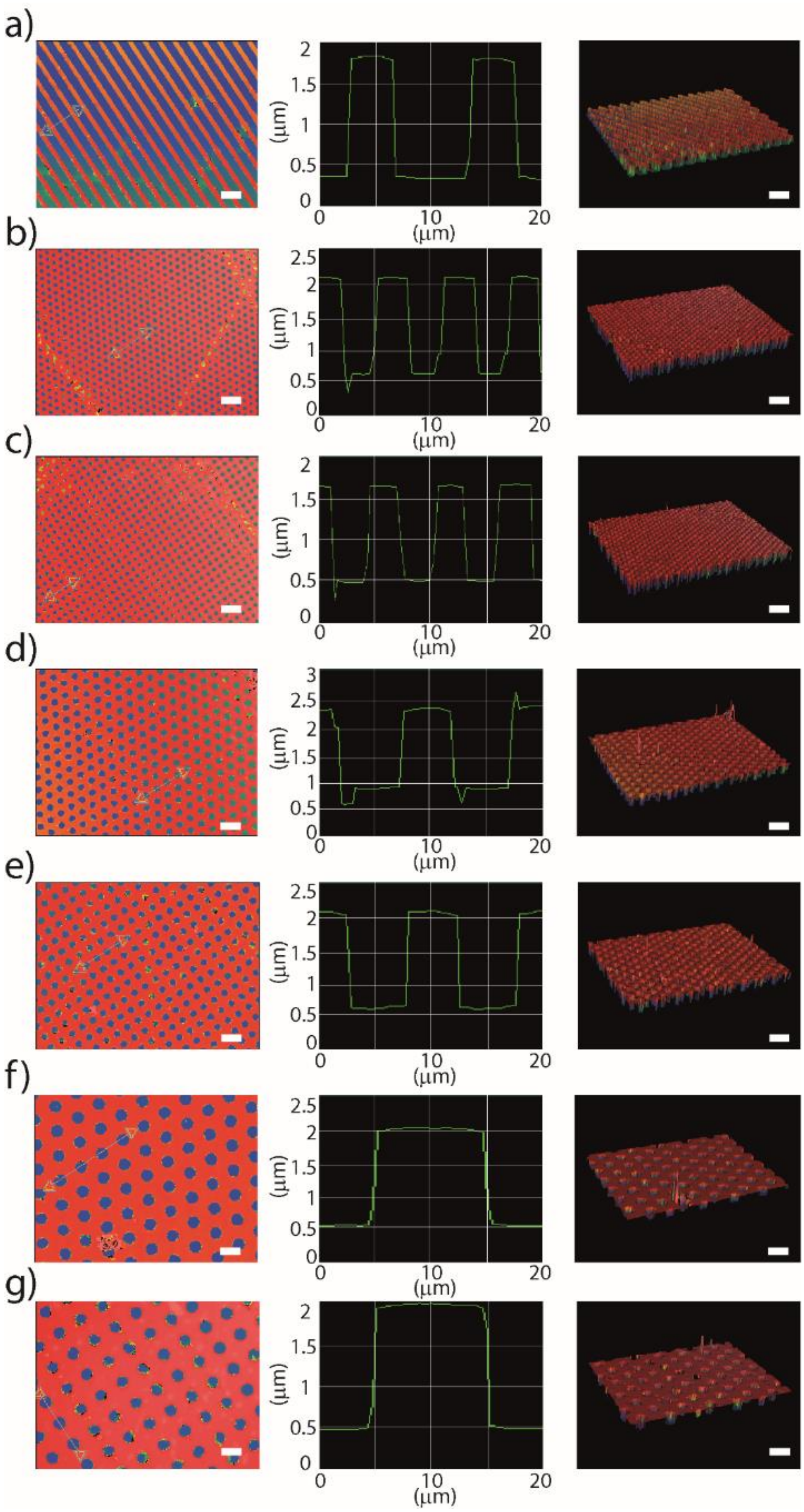

Supplementary Figure 1: <u>Optical characterization of PDMS molds</u> for *in-vitro* experiments. Scale bar: 15 µm.

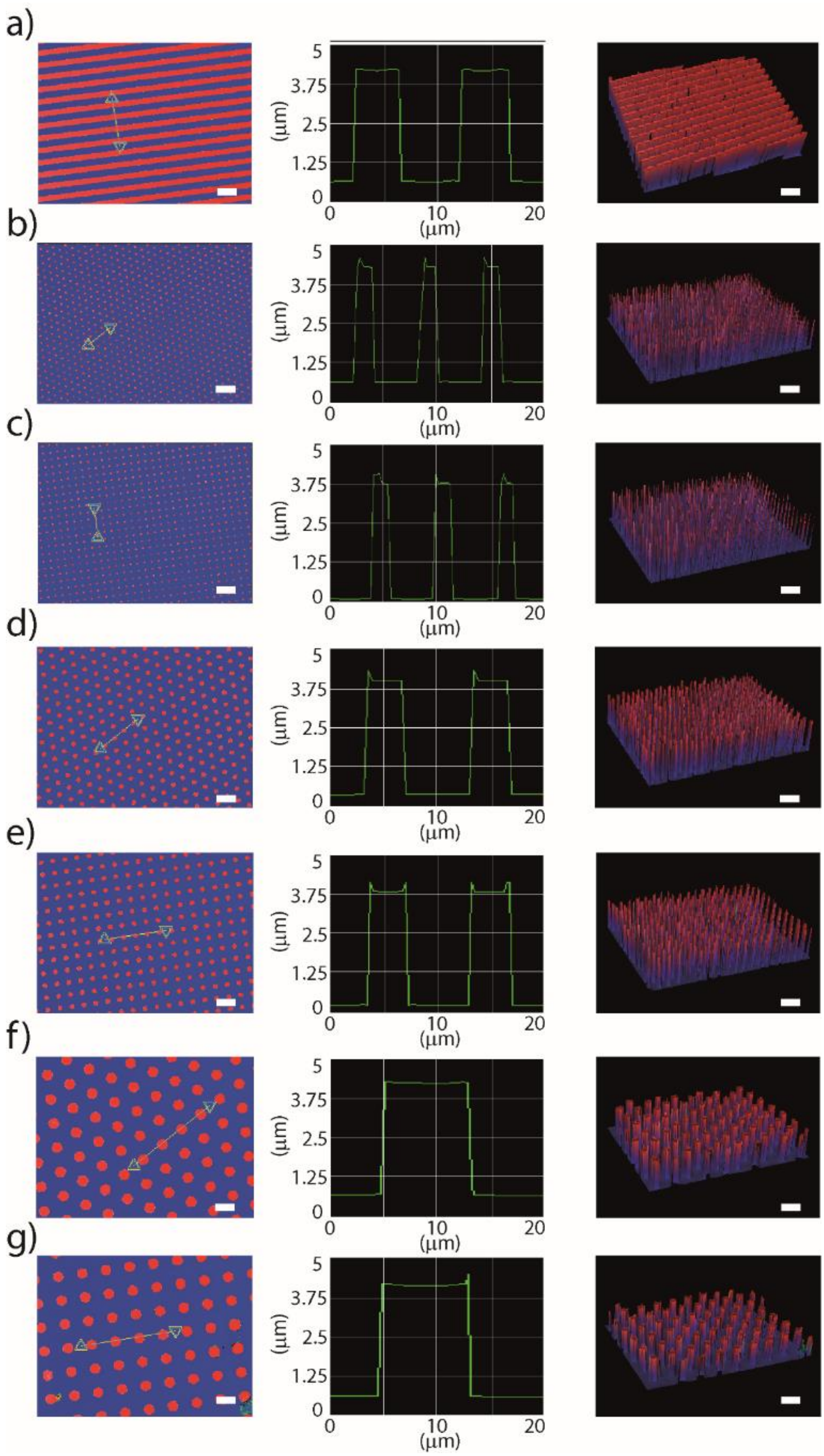

Supplementary Figure 2: Optical characterization of the PDMS molds for Guided Assembly Biolithography. Scale bar: 15 µm.

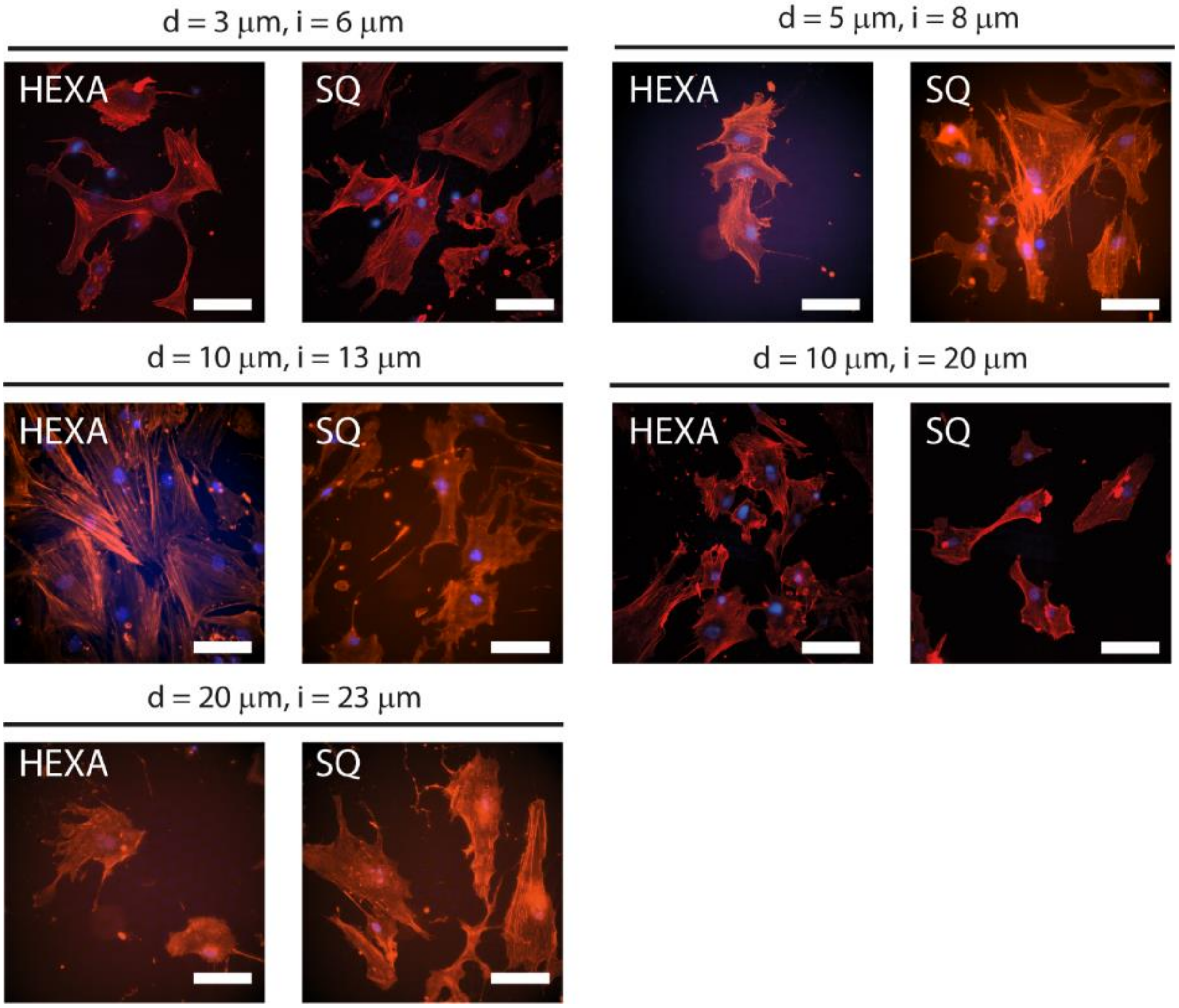

Supplementary Figure 3: Representative fluorescence images of HDFs on different patterns revealing F-Actin (red) and nuclei (blue). Scale bar: 80 μm.

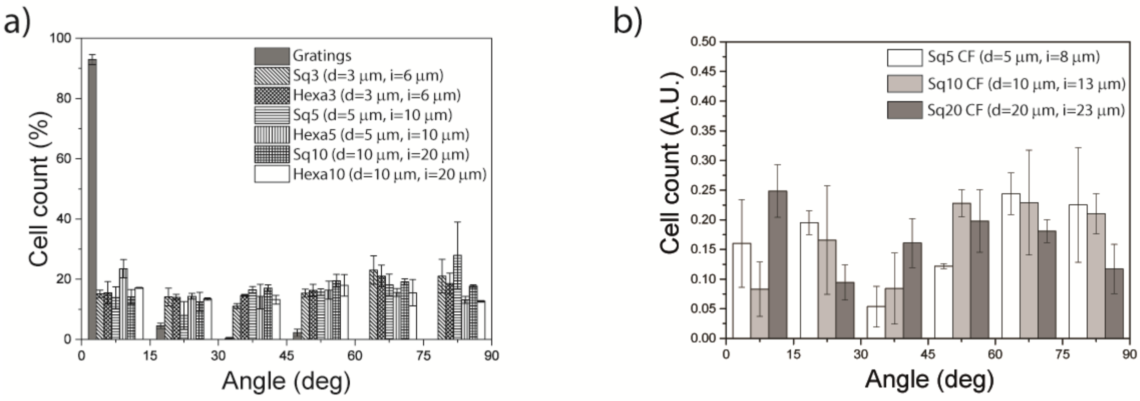

Supplementary Figure 4: **<u>Cell directionality on surface-structured PDMS.</u>** **PDMS substrates with a) constant or b) varying contact factor (CF). n=3, n'=6300. n= number of experiments, n'= number of measurements.**

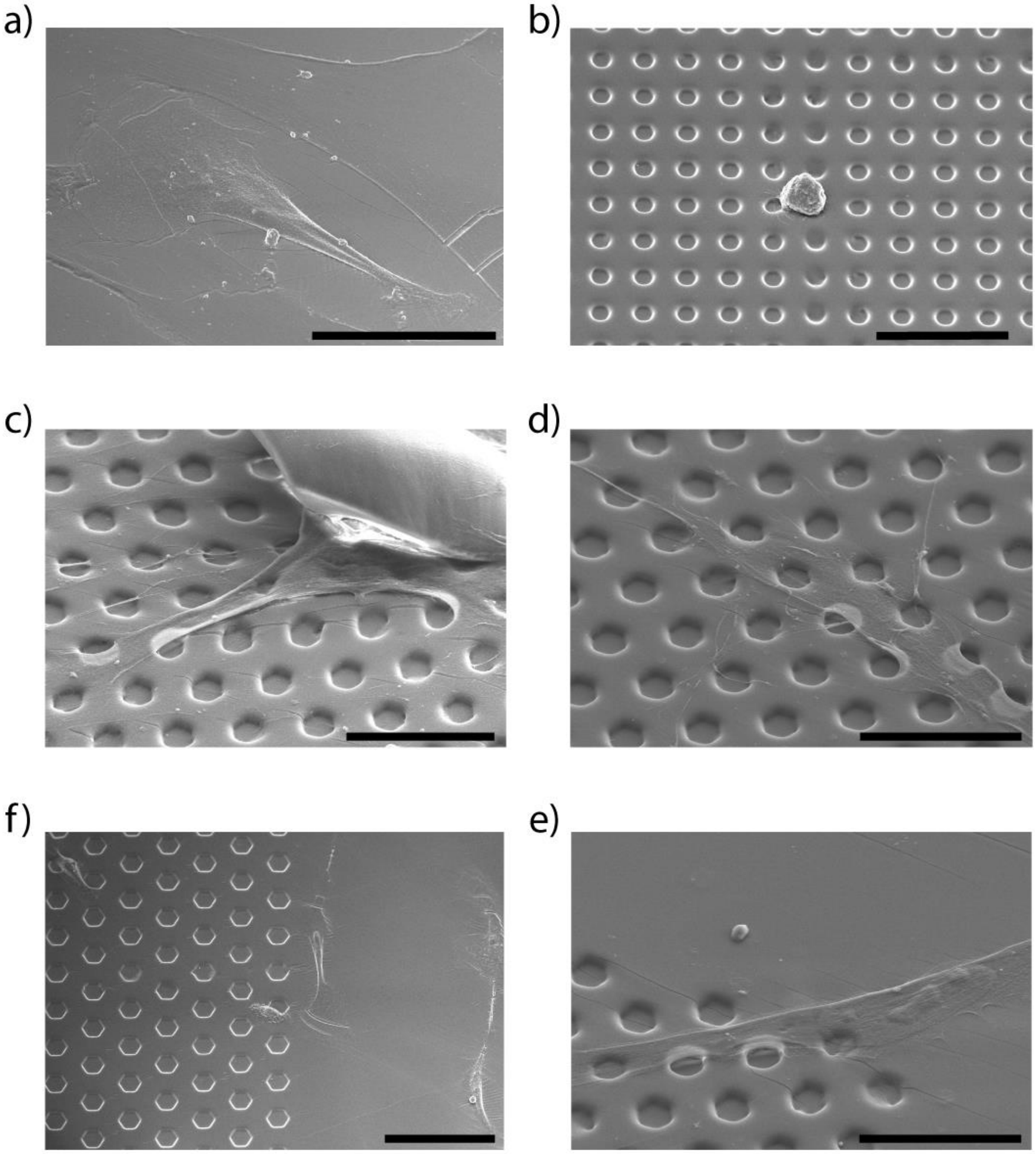

Supplementary Figure 5: **<u>Representative SEM images of HDFs morphology on PDMS substrates.</u>** **a) Reference morphology of HDFs on unstructured PDMS. b) Extreme case of round morphology of HDFs on microstructured substrate (Hexa 3, d=3 µm, i=6 µm). c,d) Representative cases of suspended cell membrane on topographic features (Hexa 5, d=5 µm, i=10 µm). e,f) Direct comparison of different morphologies on unstructured and microstructured PDMS. Scale bar b,c,d,e): 20 µm. Scale bar a,f): 50 µm.**

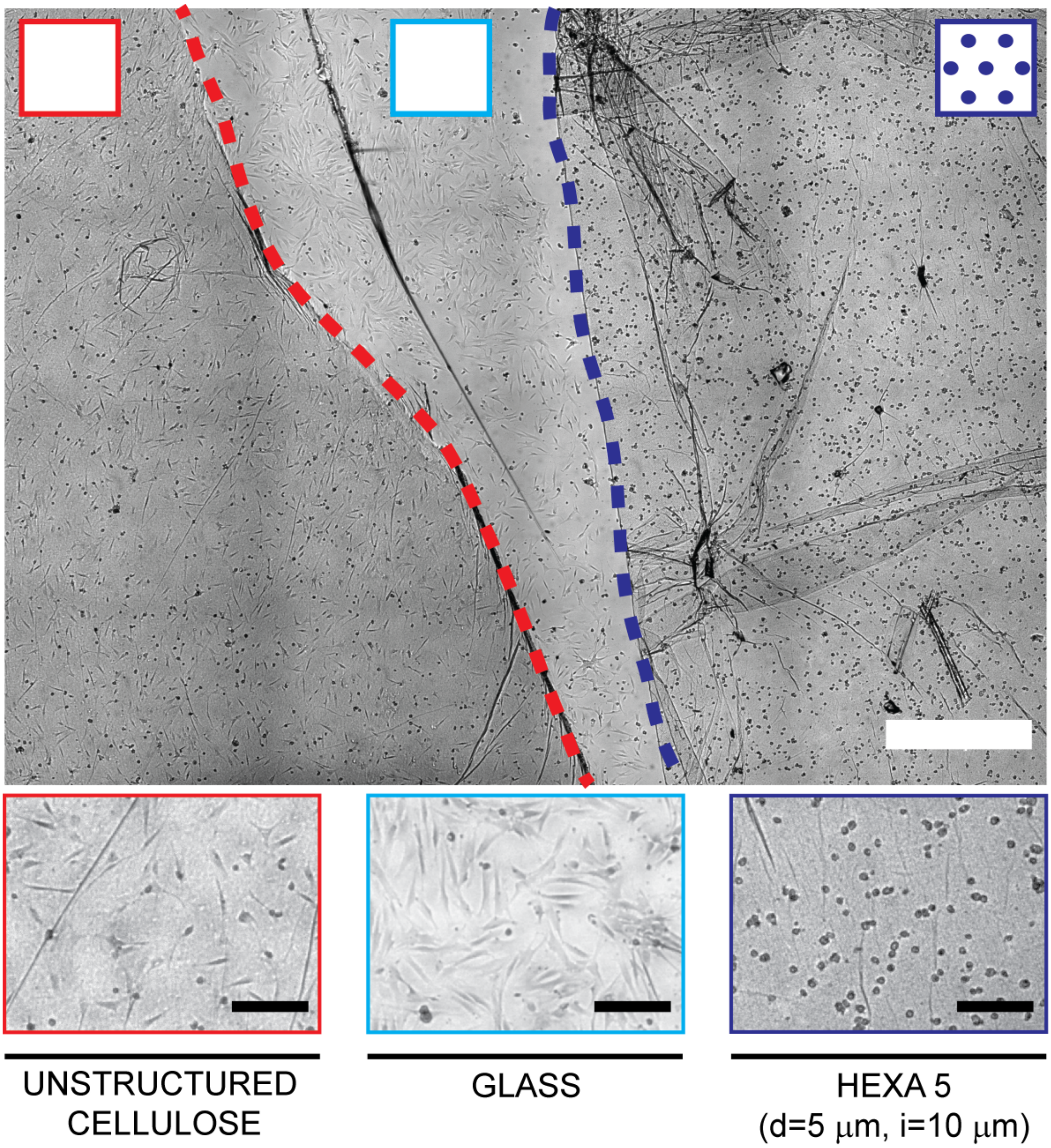

Supplementary Figure 6: **<u>Representative widefield image of HDFs morphology on different substrates.</u>** **Insets: magnified details of the three different areas in the overall image. Red, cyan and blue represent unstructured cellulose, glass and microstructured cellulose (Hexa5, d=5 μm, i=10 μm), respectively. Scale bar large image: 1 mm. Scale bar insets: 200 μm.**

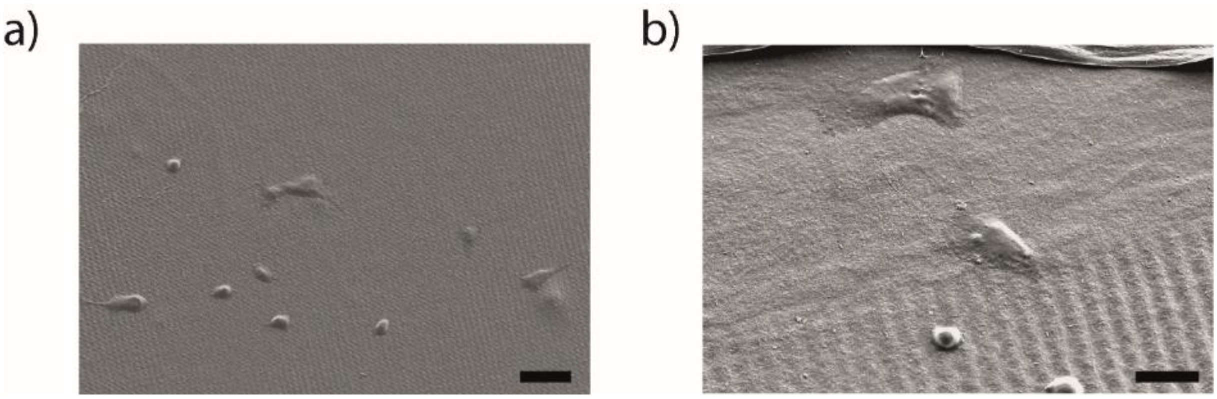

Supplementary Figure 7: **<u>SEM micrographs depict fibroblasts morphology</u> on a) unstructured and b) microstructured (Hexa5, d=5 µm, i=10 µm) biocellulose surfaces. Scale bar: a) 50 µm b) 25 µm.**

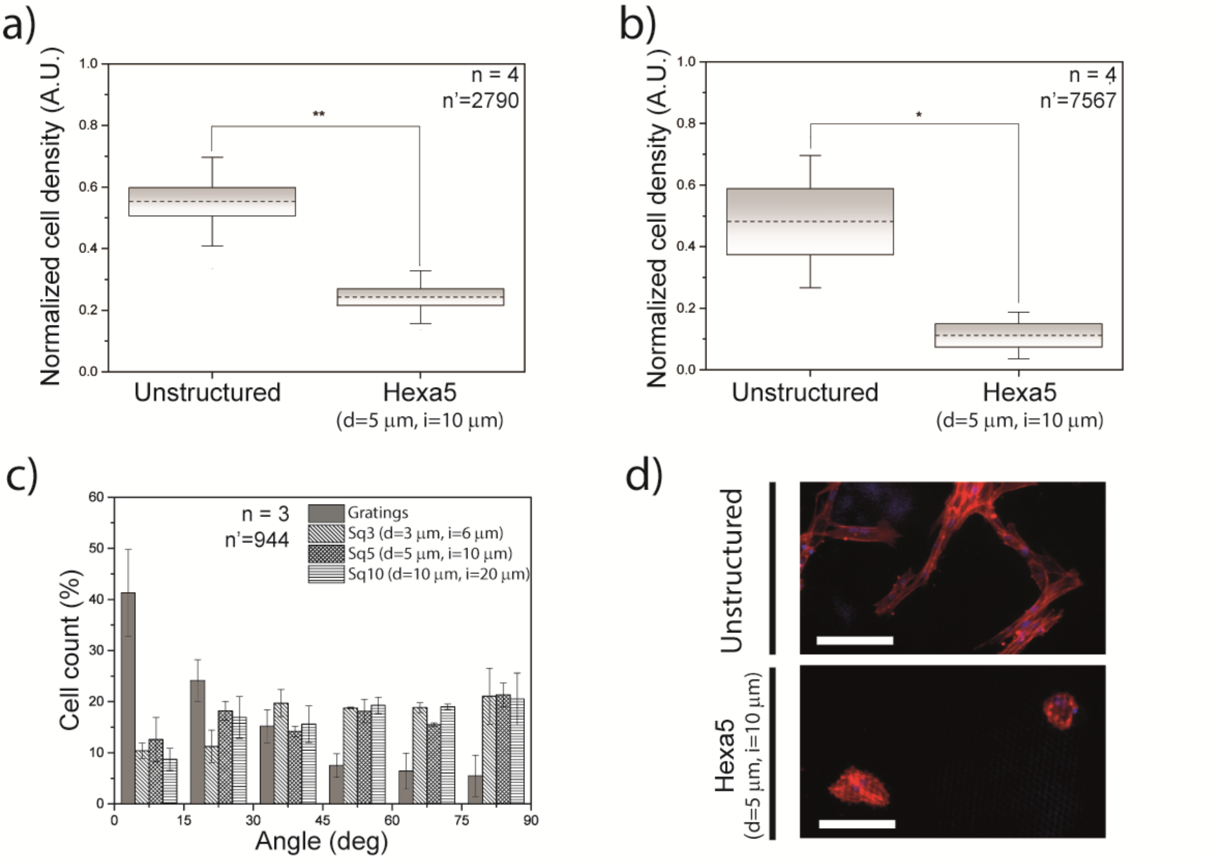

Supplementary figure 8: <u>HDFs adhesion on cellulose</u>. a) HDF density on unstructured and Hexa 5 samples normalized to cell density on surrounding glass support after 24 h of culture. b) HDF density on unstructured and microstructured (Hexa5, d=5 µm, i=10 µm) samples normalized to cell density on surrounding glass support after 1 week of culture. c) Alignment of HDFs to main directions in the underlying pattern. d) Representative fluorescence images of actin cytoskeleton on unstructured and Hexa 5 biosynthesized cellulose samples. Scale bar: 100 µm. n= number of experiments, n'= number of measurements.

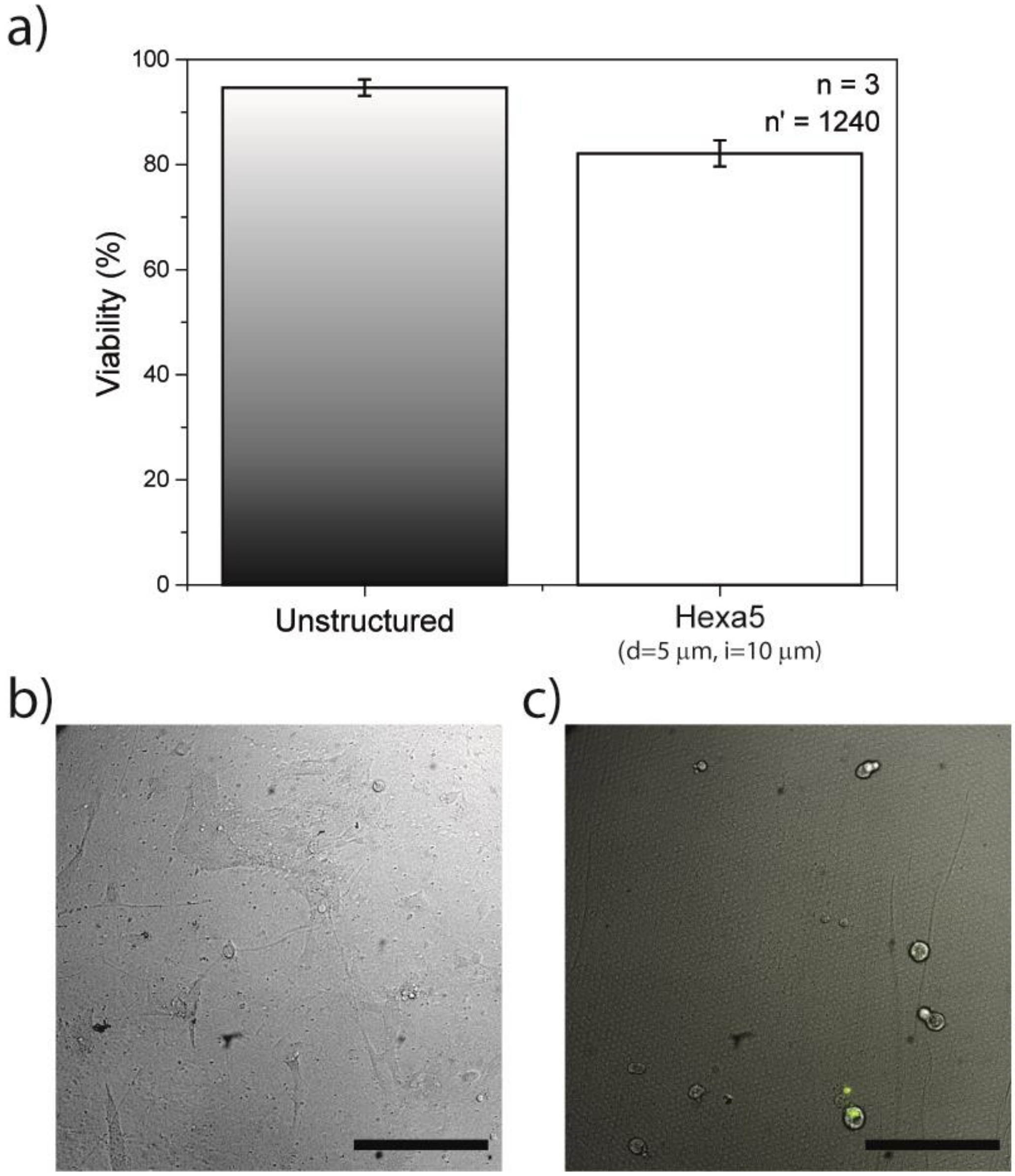

Supplementary Figure 9: **<u>Viability of HDFs on flat and microstructured biocellulose samples.</u>** **a) Percentage of viable HDFs after 72 h of culture on unstructured and micropatterned biocellulose membranes. Phase contrast image and green fluorescent image overlay of HDFs on b) unstructured and c) micropatterned biocellulose. Green fluorescent signal indicates compromized cell membrane and therefore it is a marker for cell death. Scale bar: 100 µm. n= number of experiments, n'= number of measurements.**

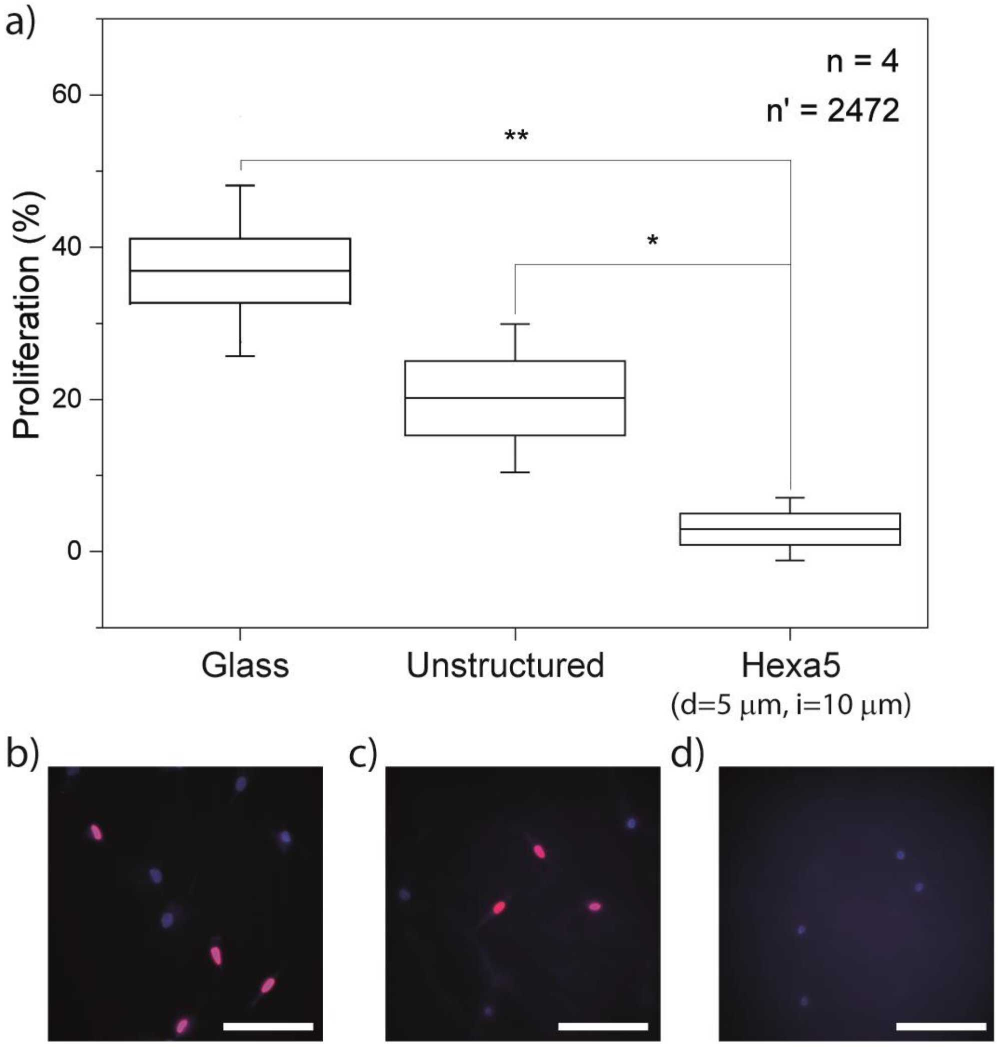

Supplementary Figure 10: <u>Proliferation of HDFs on glass, unstructured and microstructured biocellulose.</u> a) Quantified proliferation after 72 h of culture. Representative fluorescent images with overlay of blue (Hoechst) and red (Alexa Fluor 547) channels of HDFs on b) FN-coated glass, c) unstructured and d) structured biocellulose. The red fluorescent dye identifies cells that have proliferated. Scale bar. 100 µm. n= number of experiments, n'= number of measurements.

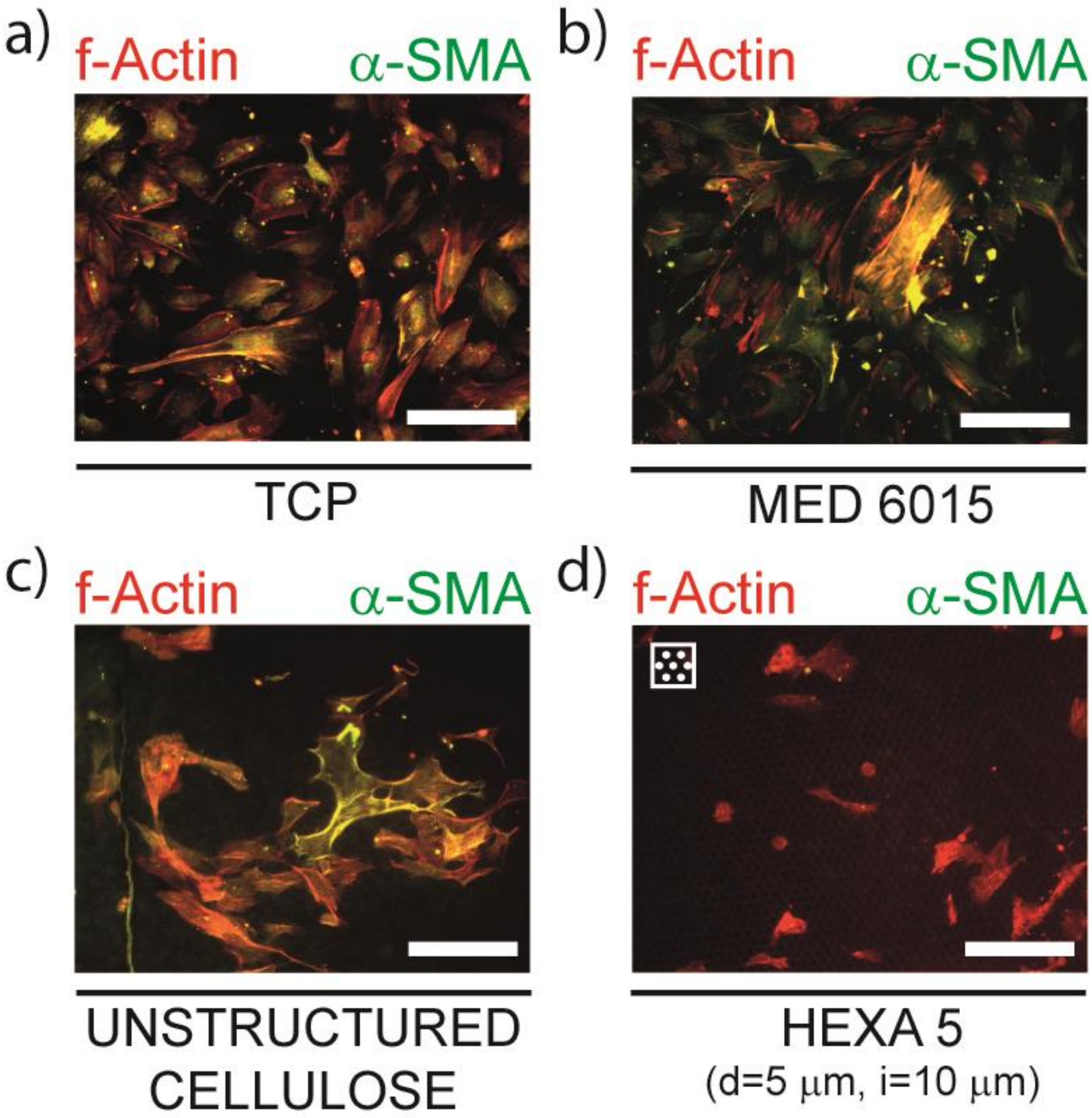

Supplementary Figure 11: <u>Immunofluorescence images of α-SMA and f-Actin</u> in fibroblasts on a) Tissue Culture Plastic (TCP), b) Medical Grade Silicone MED6015, c) unstructured biocellulose and d) microstructured (Hexa5, d=5 µm, i=10 µm) biocellulose. Scale bar: 200 µm.

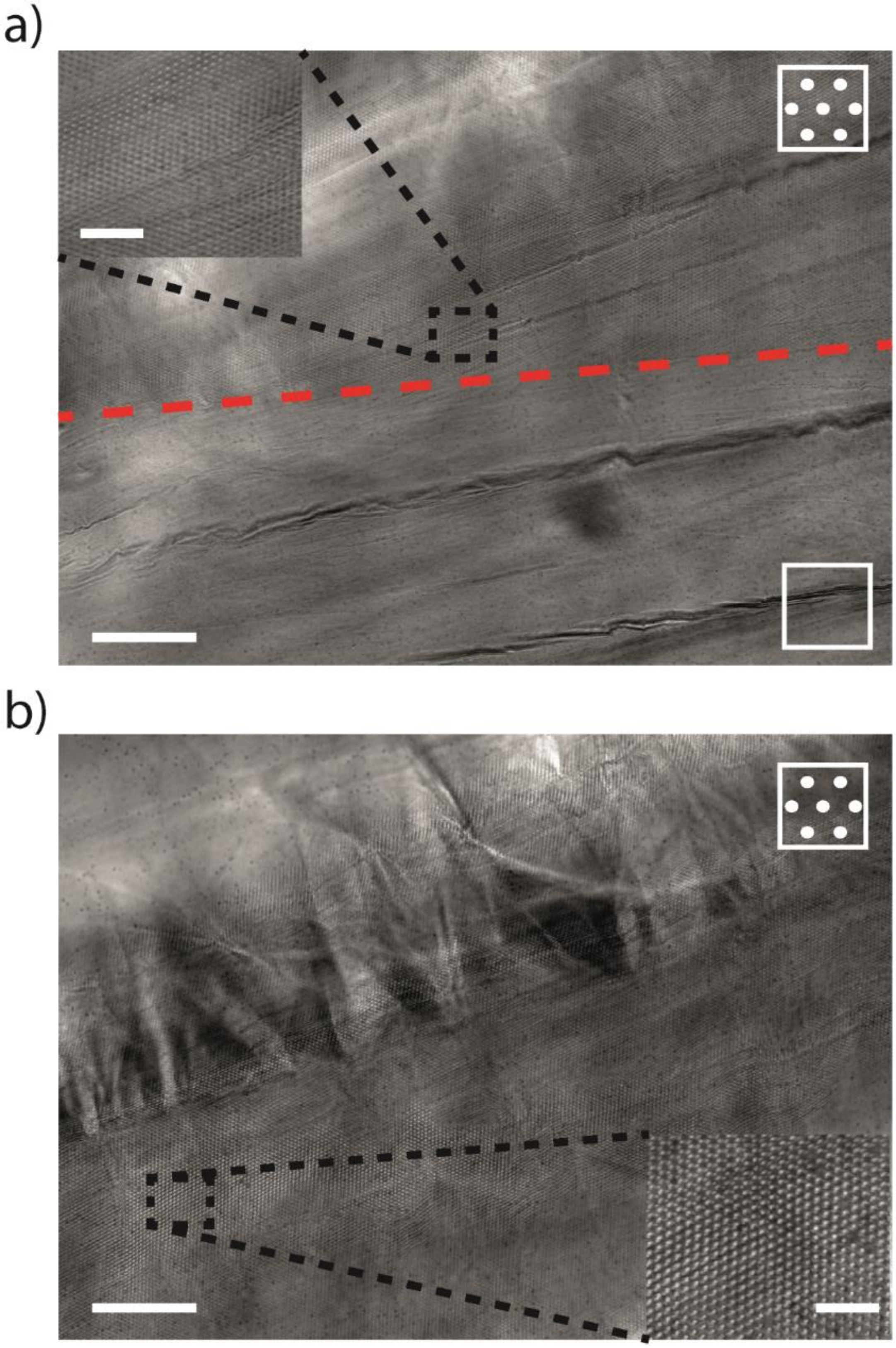

Supplementary Figure 12: **<u> Optical images of microstructured areas on samples implanted for 13 weeks in pigs.</u>** **a) Border region between the microstructured and unstructured areas of the cellulose membrane. b) Extensive area with visible microstructures. Scale bar: 200 µm. Inset: 30 µm.**

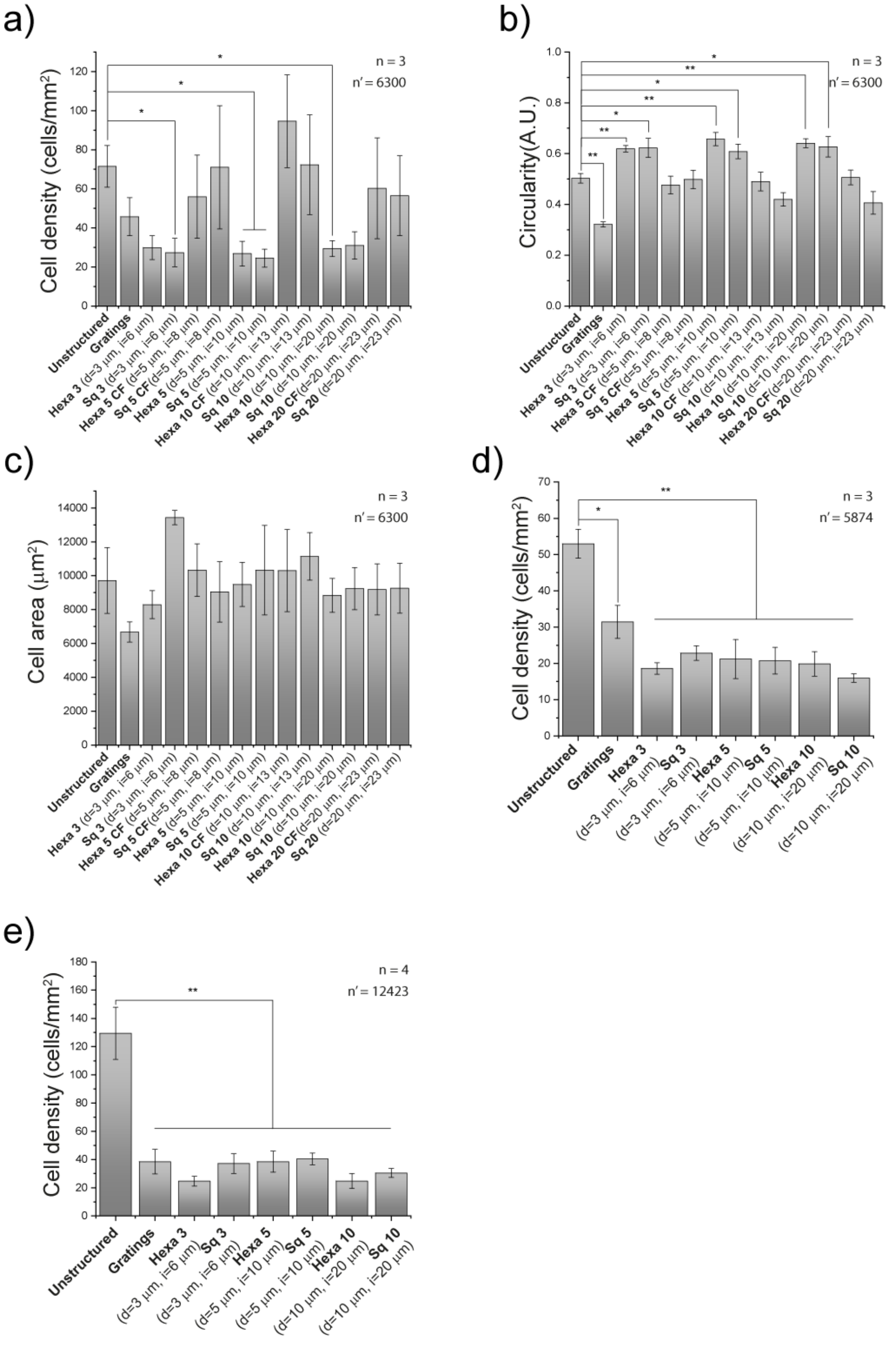

Supplementary Figure 13: **<u>Absolute values of morphometric quantities measured on different substrates.</u>** **a) HDFs density on PDMS substrates. b) HDFs circularity on PDMS substrates. c) HDFs area on PDMS substrates. d) HDFs density on biocellulose substrates. d) THP-1s density on biocellulose substrates. *p<0.05, **p<0.01 calculated with two-samples t-Student's test. n= number of experiments, n'= number of measurements.**

Supplementary Tables

Supplementary Table 1: Surface topographies with varying contact factor (CF).

Topography	***Design***		***Contact Factor (CF)***
	d_{mold} (µm)	i_{mold} (µm)	(%)
Square 5_CF	5	8	77.4
Hexa 5_CF			70.7
Square 10_CF	10	13	65.8
Hexa 10_CF			55.6
Square 20_CF	20	23	56.3
Hexa 20_CF			43.3
